# Knowledge sharing and discovery across heterogeneous research infrastructures

**DOI:** 10.12688/openreseurope.13677.1

**Published:** 2021-06-14

**Authors:** Siamak Farshidi, Xiaofeng Liao, Na Li, Doron Goldfarb, Barbara Magagna, Markus Stocker, Keith Jeffery, Peter Thijsse, Christian Pichot, Andreas Petzold, Zhiming Zhao

**Affiliations:** 1 MultiScale Networked Systems (MNS), University of Amsterdam, Amsterdam, Netherlands, 1098 XK, The Netherlands; 2 Environment Agency Austria, Vienna, Austria; 3 TIB – Leibniz Information Centre for Science and Technology, Hannover, Germany; 4 British Geological Survey, London, UK; 5 MARiene Informatie Service, Nootdorp, The Netherlands; 6 French National Institute for Agriculture, Food, and Environment, Paris, France; 7 Forschungszentrum Juelich GmbH, Jülich, Germany

**Keywords:** Knowledge base, knowledge management, search engine, research infrastructure, software development lifecycle

## Abstract

Research infrastructures play an increasingly essential role in scientific research. They provide rich data sources for scientists, such as services and software packages, via catalog and virtual research environments. However, such research infrastructures are typically domain-specific and often not connected. Accordingly, researchers and practitioners face fundamental challenges introduced by fragmented knowledge from heterogeneous, autonomous sources with complicated and uncertain relations in particular research domains. Additionally, the exponential growth rate of knowledge in a specific domain surpasses human experts’ ability to formalize and capture tacit and explicit knowledge efficiently. Thus, a knowledge management system is required to discover knowledge effectively, automate the knowledge acquisition based on artificial intelligence approaches, integrate the captured knowledge, and deliver consistent knowledge to agents, research communities, and end-users. In this study, we present the development process of a knowledge management system for ENVironmental Research Infrastructures, which are crucial pillars for environmental scientists in their quest for understanding and interpreting the complex Earth System. Furthermore, we report the challenges we have faced and discuss the lessons learned during the development process.

## 1 Introduction

Due to population growth and economic development, human impacts on natural resources are continuing to grow. Nonetheless, given the increasing complexity and capital intensity of our society and economies, natural processes in the solid Earth, atmosphere, ecosphere, terrestrial, and marine realms have an intensifying impact on humanity and society. Understanding and quantifying these pressures and resulting changes is a requirement for our societies’ sustainable development using fact-based decision-making ^1^.

Climate change, for example, has been identified as a major environmental problem for humanity by the United Nations and the European Union. Research is expected on potential scenarios on climate change that will drastically affect natural ecosystems, plants, habitat, and animals, contributing to speedup in biodiversity loss in some areas. The impacts would have knock-on effects for many communities and sectors that rely on natural resources, including agriculture, fisheries, fuels, tourism, and water. Additionally, the ocean plays a central role in regulating the Earth’s climate ^2^. As the International Oceanographic Data and Information Exchange (IODE) ^3^ has announced: "The efficient collection, integration, and use of ocean observations gathered by countries around the world for a variety of purposes requires timely, open, and unrestricted international exchange of oceanographic data. Weather and climate prediction, operational forecasting of the marine environment, life protection, mitigation of human-induced changes in the marine and coastal environment, and the development of scientific knowledge that makes this possible are examples of such purposes.".

 Assessments of climate change and their association with the driving forces must be based on trustworthy and well-documented observations. This is a difficult task due to the many interactions that exist between the atmosphere, soil, and hydrosphere. The resulting impacts on ecosystems all need particular and focused high-quality long-term observations. This forces us to have better observations and data on these essential pre-conditions to inform decision-makers better to take the measures necessary to maintain a thriving society ^1^.

Research Infrastructures (RIs) are vital for providing the required information to support science and fact-based policy development. Research infrastructures, including advanced computing and storage infrastructure, in environmental science are essential requirements for scientists in this domain to understand and analyze the sophisticated earth system ^4^. Interdisciplinary research communities and research infrastructures collaborate with the neighboring disciplines, namely atmosphere, biosphere, hydrosphere, and geosphere. Internal cooperation across different realms resulted in the formation of distinct research traditions, skills, and cultures. The interconnected essence of the earth system, on the other hand, requires the scientific community to transcend well-established divisions between disciplines and domains and work toward a common understanding of the world as a whole ^5^.

The data from the Integrated Carbon Observation Network (ICOS) ^6^ Research Infrastructure, for example, aids climate science by informing scientists and the general public on natural and human-caused greenhouse gas emissions and uptake from the ocean, land ecosystems, and atmosphere. It gives access to high-quality data processed by the Thematic Centers as raw, near real-time, and final quality-controlled data and supplemented with elaborated (model) data and analyses, almost always licensed under a CC4BY license. The IAGOS ^7^ research infrastructure provides atmospheric composition information, including greenhouse gas observations from commercial aircraft. IAGOS data are used by researchers worldwide for process studies, trend analysis, validation of climate and air quality models, and spaceborne data retrievals validation. Aerosols and their precursors are monitored by the ACTRIS ^8^ research infrastructure. Aerosols have a big impact on the Earth’s radiation balance, and consequently the climate. Their levels are inextricably linked to human activity and emissions. These infrastructures are part of a larger worldwide effort to advance science-based, high-quality observations that will help people make better decisions. As a result, the data and procedures are based on international, typically community-based standards.

Typically, RIs are domain-specific and are not connected, so that interoperability can be a critical issue for scientists involved in interdisciplinary research projects. Moreover, the researchers/developers are not knowledgeable in all domains, so a knowledge management system is required to capture knowledge automatically and enable researchers to access data, software tools, and services from different sources and integrate them into cohesive experimental investigations with well-defined, replicable workflows for processing data and tracking results’ provenance. Accordingly, a knowledge management system is required for research communities that (1) discover knowledge and capture them automatically, (2) answer any domain question without any limitation to its current search space, (3) deal with noisy sets of retrieved documents, likely consisting of many irrelevant documents and semantically and syntactically ill-formed documents, (4) have an advanced search engine to interpret and reformulate queries by information retrieval algorithms, (5) return a set of recommended solutions (answers) based on the retrieved documents, and (6) visualize its outcomes to facilitate the data analysis for research communities. This paper introduces a novel Knowledge management system, called ENVRI-KMS, to meet the ENVRI research community’s requirements and make the research assets Findable, Accessible, Interoperable, and Reusable (FAIR ^9^) for the community.

The rest of this study is structured as follows: Section 2 introduces knowledge discovery and sharing challenges, formulates the research questions, and elaborates on the research methods that have been employed to capture knowledge regarding the ENVRI-KMS. Section 3 positions the proposed approach in this study among the other knowledge management approaches in the literature.

Section 4 outlines the development process of the ENVRI-KMS. Section 4.1 explains the online survey that we conducted to collect requirements of the ENVRI-KMS. Section 4.2 shows the use case scenarios that we identified based on the survey. Section 4.3 introduces the design decisions that we made to design the ENVRI-KMS architecture. Section 4.4 reports the results of the documentation analysis that we have done to compare potential technologies in the market that can be employed to implement the ENVRI-KMS. Section 5 elaborates on the selected technologies that we employed to develop the ENVRI-KMS and demonstrates part of the current implementation. Section 6 analyzes the requirements and maps them to the survey questions and research questions based on the participants’ responses. Section 7 highlights the challenges and lessons learned during the development process of the ENVRI-KMS. Finally, Section 8 summarizes the proposed approach, defends its novelty, and offers directions for future studies.

## 2 Challenges regarding knowledge sharing and discovery

A significant number of advanced research support environments, such as ICOS ^6^ are IAGOS ^7^, are available to facilitate the access of researchers to research assets (e.g., data products, best practices, data service design decisions, software tools, and services). Such research assets are scattered among a wide range of heterogeneous knowledge resources ^4^. Furthermore, operational policies of different domains typically restrict interoperability and accessibility of multidisciplinary research projects. Additionally, technical reports about architectural design, service interfaces, selections of metadata standards, controlled vocabularies, and ontologies are not shared effectively. Accordingly, the main research question in this study is *"How to enable a domain-specific research community with their asset discovery challenges based on the FAIR principles?"*

As knowledge is scattered in a wide range of literature, forums, documentation, and tacit knowledge of domain experts, the following research questions should be addressed to capture knowledge systematically: *RQ*_1_: Which sources of knowledge should be employed to build the knowledge management system’s search space? *RQ*_2_: How to capture knowledge systematically? *RQ*_3_: How to keep the knowledge base always up to date? *RQ*_4_: How to store and retrieve acquired knowledge when it is needed? *RQ*_5_: How to evaluate the recommended solutions of the knowledge management system?

This study employs a mixed research method based on design science research, surveys, and documentation analysis to capture knowledge regarding knowledge management systems and answer the research questions. The research approach for creating the proposed knowledge management system, called ENVRI-KMS, is Design Science, which addresses research by building and evaluating artifacts to meet identified business needs ^10^ in an iterative process ^11^. Furthermore, we designed a survey form and asked several of our colleagues to critique it. We conducted an online survey in the context of 26 research infrastructures to collect their functional requirements and quality concerns. In total, 35 domain experts participated in the research to assist us with the ENVRI-KMS development life cycle and the requirements elicitation phase. Moreover, to develop the ENVRI-KMS, we reviewed webpages, whitepapers, scientific articles, fact sheets, technical reports, product wikis, product forums, product videos, and webinars to collect data. A structured coding procedure is employed to extract knowledge from the selected sources of knowledge.

Knowledge management systems employ problem-solving techniques, and knowledge discovery approaches to answer particular questions ^12, 13^. Knowledge discovery is the process of extracting useful and hidden information ^14^. A variety of Knowledge management systems have been introduced in literature ^15– 17^. Most of the existing knowledge management systems in the literature bound to a limited search space and optimized to address questions in a particular context. Each question-answer-context tuple is well-formed, standardized, and generated rising from the context in which the question and answer were extracted.

In this paper, we present a novel knowledge management system, called ENVRI-KMS, to meet the ENVRI research community requirements and make the research assets Findable, Accessible, Interoperable, and Reusable (FAIR ^9^) for the community. The ENVRI-KMS is a Knowledge-as-a-Service for the RI development communities to document RI services’ development and operation and address engineering problems. More specifically, the knowledge management system should (1) ingest technical results from ENVRIplus, FAIR assessment ^1^, the key sub-domains, and other tasks using a formal language for knowledge representation and proven semantic technologies; (2) provide services and tools to enable RI developers and data managers to browse, search, retrieve and compare RI technical statuses and technical solutions to development problems via available content; (3) provide content management tools for specialists in the ENVRI community to ingest new knowledge and control the quality of content; (4) also provide interfaces to other existing semantic resources, e.g., the service catalog of a future ENVRI-HUB ^2^, to enhance knowledge discovery and cross-RI search, between knowledge services and the online presence of ENVRI resources.

## 3 Related work

We realized that researchers in the literature have introduced a variety of tools and techniques to address knowledge management challenges. A subset of selected studies is presented as follows. Note, we categorized the selected studies into “ *Knowledge management development*” approaches (research papers) and “ *Knowledge management systems*” (tool papers). Table 1 shows the key factors of the selected studies and compares them against this study. The table shows that our research methods in this study are literature study, document analysis, survey, and design science.

**Table 1.** The results of the systematic literature review based on Snowballing (citation tracking) are presented here. The table shows the comparison of the selected studies and this study against a set of key factors, including research methods, publication types, research types, emphasized lifecycle phases, and contexts.

Study	Year	Research Method	Publication Type	Research Type	Lifecycle Phase	Context
This study	2021	Literature Study Document Analysis Survey Design Science	Research Paper	Conceptual Operational	Planning Requirement Elicitation Architecture Design Implementation	Knowledge Engineering Knowledge Management Knowledge Discovery Knowledge Acquisition Knowledge Representation
18	1992	Literature Study	Research Paper	Conceptual	Architecture Design	Knowledge Engineering Knowledge Acquisition
19	2001	Literature Study	Research Paper	Conceptual	Architecture Design	Decision-Making Process
20	2019	Literature Study	Research Paper	Conceptual	Planning	Knowledge Management
21	2018	Survey	Research Paper	Conceptual	Planning Requirement Elicitation	Knowledge Management
22	2002	Literature Study	Research Paper	Conceptual	Planning	Knowledge Management
23	2005	Literature Study	Research Paper	Conceptual	Maintenance	Knowledge Management
24	2020	Literature Study Experiment	Research Paper	Conceptual Operational	Architecture Design Implementation	Knowledge Discovery Knowledge Representation
25	2017	Literature Study	Research Paper	Conceptual	Planning	Knowledge Management
26	2019	Case Study	Research Paper	Conceptual	Planning	Knowledge Management
27	2019	Case Study	Research Paper	Conceptual	Planning	Knowledge Engineering Knowledge Management Knowledge Discovery
28	2018	Literature Study	Research Paper	Conceptual	Planning	Knowledge Management Decision-Making Process
29	2019	N/A	Tool Paper	Operational	Implementation	Knowledge Discovery
30	2017	Literature Study	Tool Paper	Conceptual Operational	Architecture Design mplementation	Knowledge Discovery Knowledge Acquisition
16	2020	Literature Study Document Analysis Design Science	Tool Paper	Conceptual Operational	Architecture Design Implementation	Decision-Making Process Knowledge Management
31	2018	Literature Study	Tool Paper	Conceptual Operational	Architecture Design Implementation	Knowledge Discovery Knowledge Acquisition
32	2002	N/A	Tool Paper	Conceptual Operational	Architecture Design Implementation	Knowledge Management
33	2006	Literature Study	Tool Paper	Conceptual Operational	Architecture Design Implementation	Knowledge Management

Additionally, this research paper’s scope is mainly on the conceptual design and implementation of a knowledge management system called ENVRI-KMS. Moreover, we reported the planning, requirement elicitation, architecture design, and implementation phases of the ENVRI-KMS. The paper’s main contexts are Knowledge Engineering, Knowledge Management, Knowledge Discovery, Knowledge Acquisition, and Knowledge Representation.

### 3.1 Knowledge management approaches

Wielinga *et al*. ^18^ explained knowledge-based systems’ development as a modeling activity. They introduced five fundamental principles underlying their approach, including (1) the introduction of partial models as a means to cope with the complexity of the knowledge engineering process, (2) a framework for modeling the required expertise, (3) the reusability of generic model components as templates supporting top-down knowledge acquisition, (4) the process of converting simplistic models into more complicated ones, and (5) the impact of the structure-preserving transformation of models of expertise on design and implementation.

Sapuan ^19^ reported a set of knowledge management systems’ architectures, concepts, and development processes. Additionally, the author highlighted the importance of knowledge-based systems in the context of concurrent engineering.

Martins *et al*. ^20^ conducted an extensive literature review to capture knowledge regarding knowledge management systems in the context of sustainability. Accordingly, they analyzed and identified actions for small and medium companies that meet sustainable development guidelines. Moreover, they explored the Universities’ possibilities in generating knowledge for the search of a more sustainable society. They highlighted studies that aimed to analyze and propose guidelines for the adequacy of productive operations to achieve sustainable goals.

Santoro *et al*. ^21^ investigated the relationship among knowledge management systems, open innovation, knowledge management capacity, and innovation capacity. They employed structural equation modeling on a sample of 298 Italian firms from various sectors. Their findings indicate that a knowledge management system facilitates the creation of open and collaborative ecosystems, exploits internal and external flows of knowledge through the development of internal knowledge management capacity, and increases innovation capacity.

Lee and Hong ^22^ define knowledge management concepts and distinguish them from business process reengineering and learning organization in terms of information technology application. They conducted an extensive literature study on how application systems support each step of a knowledge management lifecycle.

Akhavan *et al*. ^23^ explained and analyzed the main failure factors of implementing a knowledge management system in a pharmacist company. They highlighted lack of top management commitment and support, improper selection of knowledge team leader and members, improper planning, lack of separate budget for knowledge management project, organizational culture, lack of cooperation between team members and employees, and resistance against the change as the key failure factors of the knowledge management system.

Castellano and Vessio ^24^ presented an approach for visual link retrieval and knowledge discovery in painting datasets. The proposed approach employs the deep convolutional neural network as a feature extractor and a fully-unsupervised nearest neighbor approach as an image retrieval system.

Iskandar *et al*. ^25^ presented an overview of the current knowledge management system issues reported in the literature by conducting a systematic literature review study on articles written during the last two decades.

Albassam ^26^ presented the findings of quantitative and qualitative studies in order to define the governance dimensions that can assist Saudi Arabia in developing an efficient knowledge management framework for long-term growth. In developing a quality information management system in Saudi Arabia, the study’s findings emphasize the importance of enhancing public sector performance, fighting corruption, and enabling rule-of-law systems.

Orenga and Chalmeta ^27^ introduced a methodology that can help companies discover, gather, manage, and apply their knowledge using Web 2.0 and Big Data tools, making the process of implementing a knowledge management system easier. An initial version of the technique was created first. An oil and gas company then used it to analyze and refine it. The results obtained show the methodology’s effectiveness.

Hellebrandt *et al*. ^28^ introduced a methodological framework based on the analytical network process (ANP) approach for selecting knowledge management solutions for complaint knowledge transfer to product development. Their framework is based on extensive literature review, competing objectives, various criteria, and various organization-specific factors.

### 3.2 Knowledge management systems

VarSome ^29^ is both an annotation tool and search engine for human genomic variants and a platform enabling the sharing of knowledge on specific variants.

Wachsmuth *et al*. ^30^ introduced a search engine framework for acquiring, mining, assessing, indexing, querying, retrieving, ranking, and presenting arguments while relying on standard infrastructure and interfaces. GIGGLE ^34^ is a genomics search engine that identifies and ranks the significance of genomic loci shared between query features and thousands of genome interval files.

Farshidi *et al*. introduced a framework and knowledge management system to build decision models for database management systems ^35^, cloud service providers ^36^, software architecture patterns ^16, 37^, model-driven development platforms ^38^, programming languages ^39^ and blockchain platforms ^40^. The authors have conducted several case studies to evaluate the decision models’ effectiveness and usefulness to address these decision-making problems.

Chantamunee *et al*. ^31^ introduced a knowledge discovery tool for searching Thai research articles in its repository. 

Chau and Chuntian ^32^ proposed a knowledge management system on flow and water quality to simulate human expertise and heuristics in problem-solving and decision-making in the coastal hydraulic and transport processes. They explained the system’s development process and illustrated a number of examples and applications of their system.

Park and Kim ^33^ proposed a framework for designing and implementing a knowledge management system for the fourth generation of Research and Development (R&D). They defined the evolutionary classification of the R&D generations and the corresponding characteristics of the respective generations.

## 4 ENVRI knowledge management system

The ENVRI-KMS is a cluster-level knowledge base that allows different ENVRI users, such as RI developers and data managers, to effectively share their technical practices, identify common data and service requirements and design patterns, and facilitate the search and analysis of existing RI solutions for environmental RI interoperability challenges ^41^.

### 4.1 Requirements elicitation

We organized an event for conducting an online survey in the context of 26 research infrastructures to collect their functional requirements and quality concerns ^42^. In total, 35 domain experts participated in the research to assist us with the ENVRI-KMS development life cycle and the requirements elicitation phase. The domain experts were selected according to their expertise and years of experience. On average, the participants had more than ten years of experience within their expertise. They were totally aware of potential challenges that researchers in their community and field might face while performing their daily tasks. Firstly, we introduced the potential functionality of the ENVRI-KMS and presented some of its applications. Then, we used an online survey tool, called Mentimeter ^43^, to distribute a virtual questionnaire including the following questions: (Q1) What information will you typically search from the ENVRI community? (Q2) What will be the typical queries you would ask the ENVRI-KMS? (Q3) How do you currently search for information from the ENVRI community? (Q4) Which knowledge management system functionality do you feel most beneficial for you? (Q5) What function do you expect from the next version of the ENVRI-KMS?

Next, we have collected all responses and prioritized them based on analyzing the frequencies of similar statements ^3^. According to their responses we extracted the following set of requirements: The ENVRI-KMS should: (R01) have all potential RIs, datasets, repositories, best practices, service catalogues, design decisions in its search space; (R02) suggest lists of contacts who are responsible for specific tasks (authors, researchers, developers, etc.); (R03) identify assessment criteria and evaluate the search space entities based on the FAIRness criteria; (R04) indicate types of search space entities, such as private, public, open-source, or premium; (R05) recommend documentation, technical solutions, configurations, and compatible combinations; (R06) offer Q&A forums for technical discussions and engage domain experts to be involved; (R07) support ontologies and semantic search; (R08) support multilingual queries; (R09) support source code search and offer relevant solutions to technical issues; (R10) be able to search RI website’s contents (similar to Google search engine); (R11) have a user interface identical to standard search engines; (R12) support SPARQL queries and be able to connect to endpoints; (R13) have high performance and availability; (R14) offer APIs to be connected to virtual research environments (ENVRI-HUB); (R15) support automated knowledge ingestion; (R16) visualize its findings and contents; (R17) support image search and be able to search multiple image categories (plots, etc.); (R18) provide assessment tools for evaluating its contents by domain experts; (R19) manual knowledge ingestion; (R20) keep its contents always up-to-date; (R21) offer geolocation of the search space entities; (R22) offer the metadata of the search space entities; (R23) support continuous integration and continuous delivery (CI/CD); (R24) offer recommendations to different user categories (researchers, knowledge curators, developers, and high-level managers); (R25) categorize and classifies its knowledge base contents.

The initial user stories for the ENVRI-KMS mainly focus on the data manager, RI service, or Virtual Research Environment (VRE) ^44, 45^ developers, e.g., for enabling a developer to check the existence or details of data management solutions from different RIs. Accordingly, the following key technical requirements have been identified to design and implement the ENVRI-KMS:

**Compatible with semantic web technologies.** As the most common type for knowledge storage, representation, reasoning, the support of Resource Description Framework (RDF) is the core requirement in the design and development of the ENVRI-KMS. This requirement can include the following specific options: RDF import/export, RDF storage, owl import, SPARQL, and GeoSPARQL support. It is acknowledged that while providing many advantages, especially in the context of integrating and operating on heterogeneous knowledge sources and of linking to existing external resources, RDF, but also the overall concept of operating on a non-monolithic set of data collections, comes with specific limitations as well, such as lack of support for referential integrity. Nevertheless, it is assumed that the ENVRI-KMS content’s nature is rather non-volatile, shifting this aspect into the background.

**Semantic search and query functionality.** An interface for searching and discovering ENVRI-KMS content should be provided; this could be the conventional keyword-based search or faceted search. A semantic search function is further expected to permit search based on ’similar’ or ’related’ terms across multiple ontologies/controlled vocabularies rather than strict adherence to a single controlled vocabulary or keyword set ^47^.

**Open and flexible knowledge ingestion.** Due to the variance of source types in the ENVRI community, various methods should be supported for knowledge acquisition, like form-based manual RDF ingestion, Questionnaire-based RDF triple generation, existing RDF integration, structured and unstructured information transformation, etc. Specific measures should be considered to facilitate non-technical users straightforwardly adding knowledge.

**Provenance and version control of the knowledge.** Considering the typical case where multiple users contribute to the ENVRI-KMS, provenance is of fundamental importance for monitoring and tracking issues, for example, enabling the third party to reproduce the scientific workflow for an authority to audit the whole process. This primarily refers to tracking individual additions, deletions, and updates and their administration, i.e., approval, rejection, and reversion.

**User-friendly and customizable user interface.** A clear and straightforward user interface is needed to fulfill their objectives, like query, semantic search. Different user interfaces should be offered to meet the requirements of the general public and professional users.

**Scaling and increasing performance.** A choice between centralized or distributed storage should be considered to tackle the growing size of the ENVRI-KMS. Also should be considered includes the dynamic resource scheduling facing concurrent search/query requests. Other features like collaborative editing are required to enable comments on contributions by other users.

**API interface.** An application programming interface (API) abstraction layer can help make knowledge accessible through applications to facilitate knowledge via APIs.

Among such technical requirements, the ENVRI-KMS should play a key role in the ENVRI communities to develop FAIR data services and share their best practices.

### 4.2 Use case scenarios

**Figure 1.**
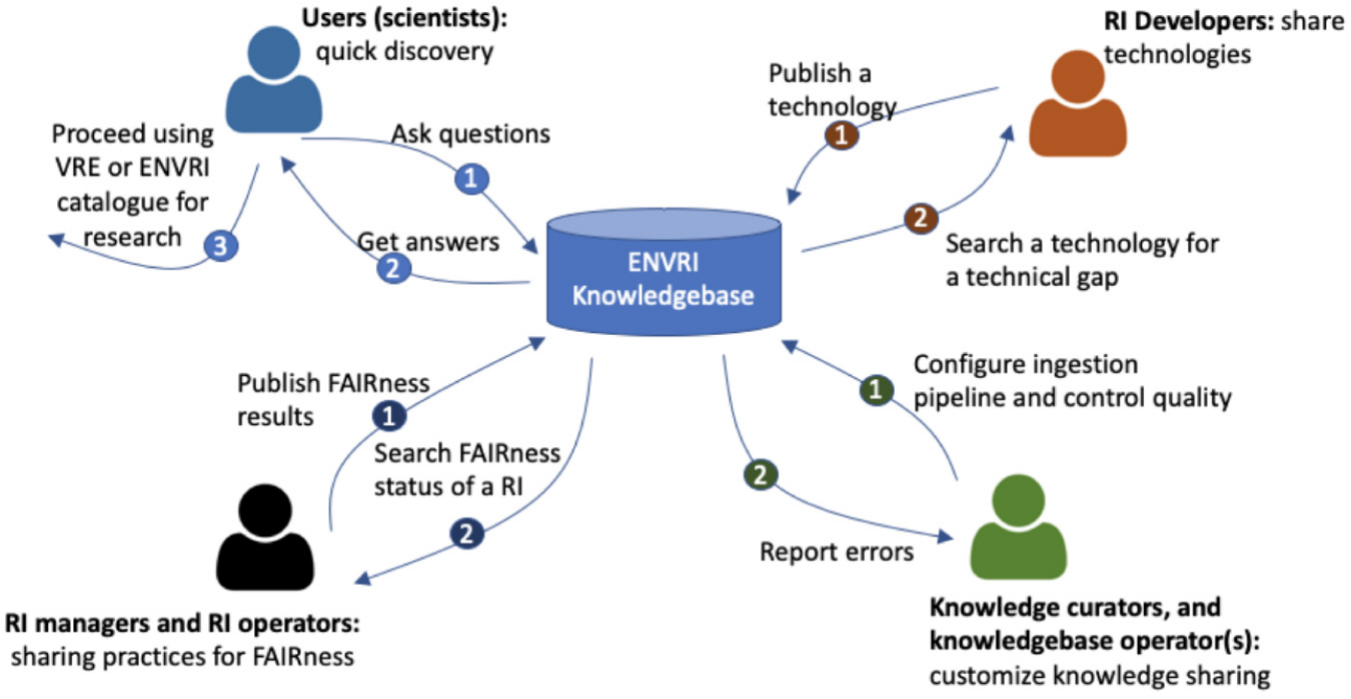
An enterprise view of the ENVRI-KMS. The enterprise view highlights the key stakeholders (namely communities in the Open Distributed Processing (ODP) term) and their interaction scenarios with the ENVRI-KMS. The numbered circles indicate the possible orders of the interactions.

Based on the survey we conducted (see Section 4.1), we identified the following four types of users (see Figure 1) of the ENVRI-KMS:

(1) **End users** may use the ENVRI-KMS to find answers to their general questions about available sources of data, services, and tools, and to use the discovered information to perform further research activities using the other tools like Virtual Research Environments or services like the RI catalogs of data or services.

(2) **RI managers** or operators may use the KB to check the status of the FAIRness of specific repositories or update the state of their RIs. The update process often needs the KB tools for FAIRness output ingestions from the other tools, e.g., the assessment wizard tool.

(3) **RI developers** may use the KB to check the existing technologies, e.g., those development results in the ENVRI portfolio or the demonstrators prepared for some known FAIRness gaps. They can also publish or update the technical descriptions using the KB tools, such as an online description form.

(4) **Knowledge curator and knowledge base operators** may use the KB to ingest content from new sources and respond to the possible errors that occurred during the ingestion or the operation.

### 4.3 Conceptual architecture

**Figure 2.**
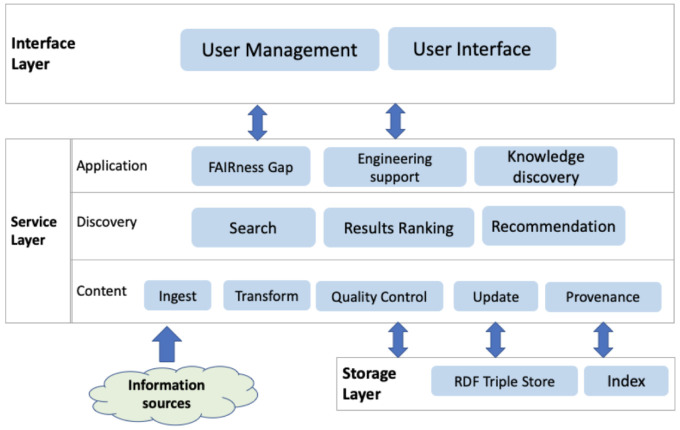
Architecture layers.

Based on the use case scenarios (see Figure 1), we design the key components of the ENVRI-KMS from the conceptual point of view. Note, the architecture is designed based on the Open Distributed Processing (ODP) framework ^48– 51^. Figure 2 shows the key components via three layers:

The **interface layer** atop contains components dealing with user-related activities. The ENVRI-KMS will be an open system for community users; the user management component is not for acquiring and processing users’ personal information but more for providing customized user support based on their interaction or contexts. A user can log in to the system using an open identity provider. The User Interface (UI) components are the application parts that allow users to interact with it. It can be formatted and rendered into various presentations to address different users’ requirements. Additionally, it validates and collects required data from users.

The **service layer** abstracts the functionality that the ENVRI-KMS offers; it can be roughly split into three sub-layers, namely:

(1) The Application sub-layer provides customized application logic (e.g., FAIRness Gap Analysis, Engineering support, or discovery knowledge from ENVRI community) based on the data passed from the underlying discovery sub-layer those results up to the User Interface Component.

(2) The Discovery sub-layer provides the functionality for searching the ENVRI-KMS, ranking the results, and recommending relevant content.

(3) The Content sub-layer provides functionality for managing the content in the ENVRI-KMS, typically in a pipeline covering: ingesting information, the transformation from information to knowledge, quality control of the knowledge generation, CRUD (Create, Read, Update, Delete) of the ENVRI-KMS content, and the provenance of these activities.

**Figure 3.**
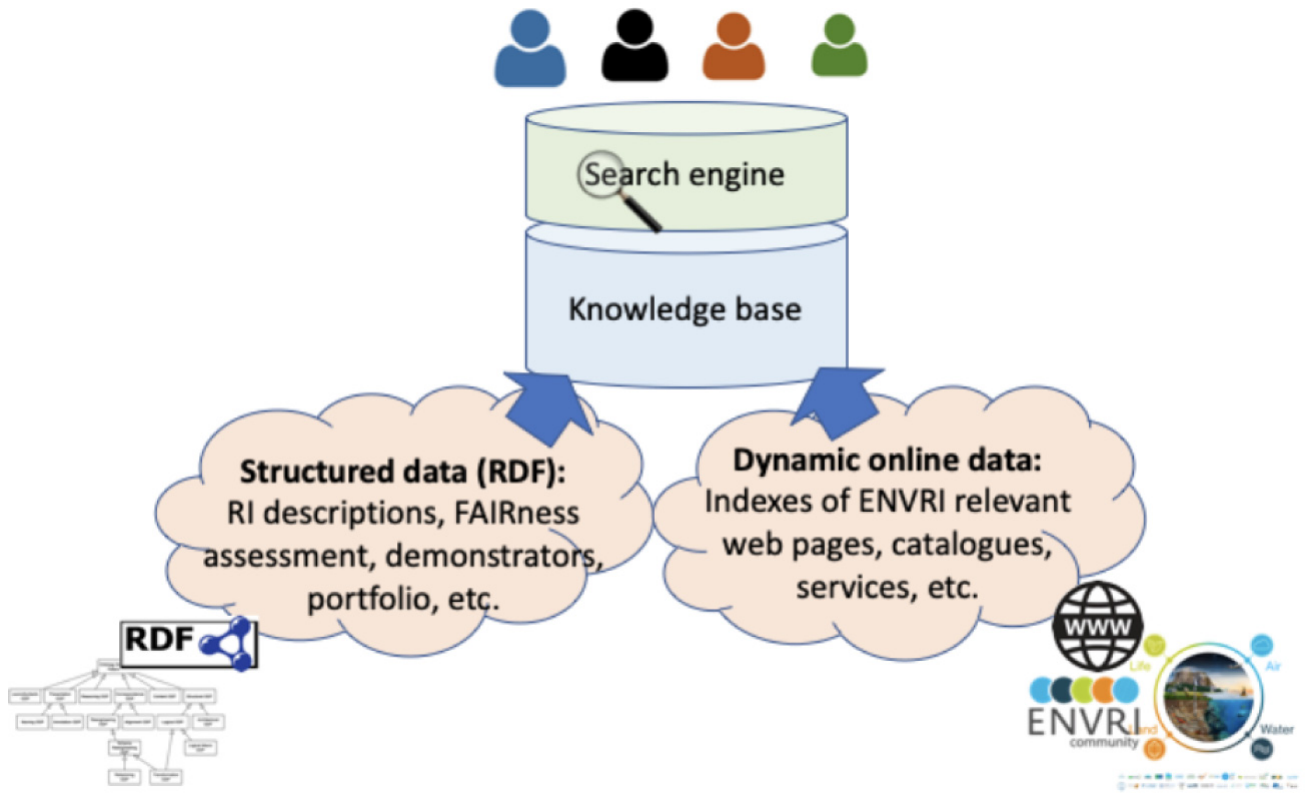
ENVRI-KMS content components. The ENVRI-KMS can be used by end users to search different contents.

The **storage layer** at the bottom is responsible for data storing and access. The data storage options needed in this project include RDF Triple Store and Inverted Index. Currently, information collected in the ENVRI-KMS consists of two main parts, as illustrated in Figure 3. The structured data in the ENVRI-KMS is based on RDF and mainly includes: (1) OIL-e (ontology of the ENVRI Reference Model) based ENVRI RI description, (2) description of the service portfolio from the previous project, and the possibly new ones in ENVRI-FAIR, (3) FAIRness principles and the results of assessing the ENVRI research infrastructures, and (4) demonstrators for tackling the known gaps, e.g., those being identified during the FAIRness assessment.

**Figure 4.**
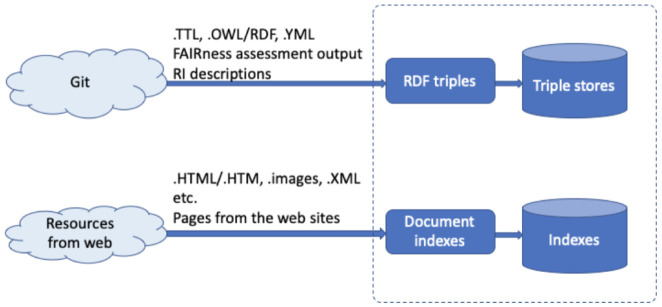
Basic information flow of the knowledge ingestion.

The versions of the structure data currently can be managed via version control systems. Currently, GitHub is used. The dynamic data in the ENVRI-KMS will be ingested from different online sources of the ENVRI communities. Figure 4 depicts the necessary information flow of the knowledge ingestion.

(1) A significant amount of KB relevant information is represented in human-readable form, residing in Wikis, other content management systems, or even static web-pages, in the "offline" text found in various documents such as books, project deliverables, or scientific publications. In the ENVRI-FAIR context, the research infrastructure websites are an excellent resource of related information, including news/events, background knowledge, etc. Similar to ENVRI, ENVRIFAIR, the community websites also contain lots of related information, like news/events, community introduction, community landscape, project information, progress, etc. These information sources have different formats, such as a webpage, word document, and pdf file.

(2) Another approach to populate the KB would be to process such free-text information to extract structured, machine-readable information. Named entity recognition would represent the first step in this regard, while the application of more complicated Natural Language Processing operations could be a valuable field of research in its own right.

(3) Information from the available catalogs of data and services. It should be clear that the indexes generated from those sources will not aim to replicate the entire catalogs but provide a quick searching capability for community users. For some RI, such information will be already managed in RDF format and accessible from triplestores.

### 4.4 Technical choices

This section reviews the relevant technologies that can be employed to develop the ENVRI-KMS according to the requirements (See Section 4.1).

***4.4.1 Data storage*.** RDF or similar triple-graph-like data structures are widely used in knowledge representation; a range of existing approaches can be used for knowledge storage, from traditional relational database management systems to dedicated graph databases.

**Relational database management systems (RDBMS)** represent the traditional approach to storing and managing structured data. The well-known MYSQL ^53^ RDBMS is, for example, used by the popular Mediawiki software to drive the Wikipedia ecosystem. Wikibase, the engine behind Wikidata, introduced in the previous section, is another example in this regard. Additional services such as the SPARQL-based Wikidata query service operate on RDF data created via MYSQL-RDF exports at regular intervals.

**Triplestores** are specialized graph databases dedicated to storing RDF data. Hybrid triplestores operate on top of traditional RDBMS, while native Triplestores provide customized data management infrastructure optimized for handling large amounts of triples. The popular Java-based RDF handling framework Apache Jena ^54^ can be operated either on a hybrid (Jena SDB) or on a native store (Jena TDB), where TDB can be operated in-memory or using a disk index. Jena Fuseki is a SPARQL server that can be run as an operating system service, a Java web application (WAR file), or as a standalone server, and it uses TDB as a transactional persistent store layer.

**Multi-model databases** act as a unified platform for storing and managing data following different paradigms. Openlink Virtuoso ^55^ is a multi-model RDBMS, data integration middleware, linked data deployment, and HTTP application server platform with great performance and scalability. In a single system, it integrates the capability of a traditional relational database management system, an object-relational database (ORDBMS), a virtual database, RDF, XML, free-text, a web application server, and a file server. The RDF engine from Virtuoso is a hybrid design that runs on top of Virtuoso’s RDBMS.

**General purpose graph databases** can handle any node-link-based information, while triplestores are designed to operate solely on RDF-based data. One fundamental distinction between RDF graphs and general-purpose graphs is that RDF does not allow for annotating individual triples (i.e., adding attributes to individual links between two instances) without relatively complicated mechanisms such as reification. So-called labeled property graphs, in turn, assign unique IDs for each link, allowing to attach attributes such as values, categories, etc. Neo4J ^56^ is an example of such a labeled property graph DB, offering a dedicated query language (Cypher) to access the stored information. In contrast to triplestores with their standard SPARQL query engines, however, there is no comparable general standard available, limiting individual solutions’ usability to the respective platforms.

***4.4.2 Knowledge base*.** Assuming RDF as a target data format for the ENVRI-KMS, this section explores solutions to allow data managers to access the ENVRI-KMS’s content using Graphical User Interface (GUI) tools to explore, search and edit the knowledge base content in a user-friendly manner. In contrast to end-user interfaces, however, the considered approaches are instead targeted at administrative tasks.

We compared five potential solutions to presenting/managing RDF-based content according to their features, such as “supporting visualization”, “The last edit on Github”, and “ Storage technology”. Besides supporting content exploration on a triple level, three of the five compared solutions also allowed for content management (upload, individual editing). The overall results of the comparison are summarized in Table 2.

**Table 2.** Comparison of existing platforms/products.

	Semantic Mediawiki	Wikibase (Wikidata)	OntoWiki
Editor	Yes	Yes	Yes
Triple Viewer	Yes	Yes	Yes
Visualization	Optional (e.g., graph extension)	Several built-in features	Optional (Cubeviz)
Original Purpose	Semantic Annotation of Wiki pages	Knowledge Base + Fact Editor for populating Wikipedia Infoboxes	Knowledge Base Editor
Full source on Github	Yes	Yes	Yes
Last Edit on Github	17.06.2019 / Active	14.06.2019 / Active	11.07.2017/Currently no further development
Frontend Technology	Browser	Browser	Browser
Server Technology	PHP	PHP	PHP
Storage Technology	Relational (can optionally be complemented with Triplestore running in parallel	Relational with mirror Triplestore for Queries	Triplestore or Relational
Native RDF	When using complementary Triplestore	No	Yes
RDF Export (Only for editor)	Yes	Yes	Yes
RDF Import	Via tools, e.g. RDFIO Tool for RDF import, convertion to internal format	Needs longer conversion workflow, would need creation of or mapping to existing Wikibase classes/properties	Yes
Builtin OWL import	No (Could be done via script, converting owl features having counterparts in internal class/property structure)	No (Could be done via script, converting owl features having counterparts in internal class/ property structure)	Yes (With limitation)
SPARQL Support	When using complementary Triplestore	Sparql queries against parallel Triplestore	Yes
Class/Category System available for instantiation	yes; proprietary; subclass/subproperty relationships possible	yes; proprietary; subclass/ subproperty relationships possible	Yes; based on OWL
Constraints (Only for Editor)	uniqueness (Property can only be used once per instance) Permitted Values (Controlled list of values to be entered for property) datatype check (One of)	Custom,	No
Provenance	Statement Level Provenance (references)	Statement Level Provenance (references)	Statement Level Provenance (references)
Revision Tracking	Yes	Yes	Yes

RDF: Resource Description Framework | OWL: Web Ontology Language | SPARQL: Protocol and RDF Query Language

**Ontowiki**, first described in 52, is a collaborative knowledge engineering platform for RDF-based data. With its latest version introduced in 14, it provides a browser-based interface for editing and browsing RDF statement collections. It puts heavy emphasis on collaborative editing features such as social comment features and statement-level provenance with history. Implemented in PHP ^4^, it can use different data backends, including relational DBMS such as MySQL or triplestores such as Openlink Virtuoso. Another flexible feature is its plugin-based architecture, enabling the addition of different features such as data visualization.

**Semantic Mediawiki (SMW)** is an extension of the popular MediaWiki software driving the well-known Wikipedia universe. It is mainly intended to augment classical Mediawiki markup pages with semantic annotation, serving, e.g., to update specific facts such as dates or quantities dynamically. Using Mediawiki as a foundation, it offers all the software’s collaboration features, i.e., discussion and version history. By default, SMW uses the standard Mediawiki data infrastructure for storage but can be extended to use a Triplestore in parallel. A significant difference to OntoWiki is that in SMW, structured information is used only for annotating entities in Wikitext and displaying lists; it is not meant to "drive" the Wiki content itself.

**Wikidata** uses a proprietary data format that provides a set of features that require workarounds to be represented as RDF, such as n-ary relations or statement-level provenance. Wikidata uses a set of Mediawiki extensions called Wikibase for its data architecture, including similar collaboration features as SMW. As described in 15, the data can be converted into an RDF representation for export, and a parallel triplestore representation of Wikidata content offers SPARQL-based querying. As of today, however, there is no means to import RDF into Wikidata directly.

***4.4.3 Search engine*.** Though some of the knowledge management systems investigated in Section 4.4.2 provide the GUI tools for querying and searing the knowledge bases, their primary target users are knowledge base administrators regarding the expertise required to use these tools. Thus, a more general user inference is still expected for a helpful search experience and context-aware exploration of the ENVRI-KMS. We investigate two systems, Open Semantic Search and Elastic Search, which provide comprehensive search solutions.

**Open Semantic Search** is an integrated tool for easier searching, monitoring, analytics, discovery, and text mining of heterogeneous and large document sets and news with free software on the user’s server.

**Elastic Search** is a search engine that is developed based on the Lucene library. It offeres a distributed, multitenantcapable full-text search engine with an HTTP web interface and schema-free JSON documents. A comparison of these two platforms in terms of our requirements is presented in Table 3.

**Table 3.** Comparison between Open Semantic Search and ElasticSearch.

	Open Semantic Search	ElasticSearch
Visualization	1. Visualizing like trend charts, word clouds, interactive maps 2. graph/network analysis view 3. Alternatively, enable the Open Source ETL plugin for integration with the Neo4J database and present visualization with Neo4j browser by Cypher graph query language.	via Kibana(Pie, Bar, Map, etc)
Builtin UI Features	Solr-PHP-UI	Search UI
Full source on Github	Yes	Yes
Frontend Technology	Browser	HTTP web interface
Storage Technology	Inverted Index	Inverted Index
Native RDF	native graph storage for graphs including RDF triple stores	index RDF data in JSON format
RDF Export (Only for editor)	Yes Yes	No
Builtin OWL import	Yes	No
SPARQL Support	Neo4j has Cypher that covers the need for a structured graph query language	Elasticsearch provides a full Query DSL (Domain Specific Language) based on JSON to define queries

***4.4.4 Knowledge graph visualization*.** We investigate several tools with network graph features for present content in the ENVRI-KMS with a knowledge graph visualization. They are:

**D3.js** ^57^ is a JavaScript library for data-driven document manipulation. Different types of data can be bound to a DOM, which may subsequently be used to perform various functions. Using the DOM data to create an SVG, canvas, or HTML visualization is one of these functions.

The most complicated component of D3 is converting the graph data into the intended map format for export (or any embeddable library that does not have a direct Neo4j connection). D3 assumes that there are two graph data collections: one for nodes and one for links (relationships). - Each of these maps has an array of characteristics for each node and relationship, which D3 converts to circles and lines.

Converting the graph data into the intended map format for export is the most difficult aspect of D3 (or any embeddable library that does not have a direct Neo4j connection). D3 assumes two graph data collections: one for nodes and another for links (relationships). - of these maps contains an array of properties for each node and relationship, which are then converted into circles and lines by d3. Force-directed graphs are now supported in D3.js versions 4 and 5, which customize the visualization to the user’s view pane.

**Popoto.js** ^58^ is one kind of tools that are embeddable tools with built-in Neo4j connections. This kind of embeddable tool can be included as a dependency within an application. It can easily be configured and styled for an application and Neo4j. This tool can easily connect to an instance of the Neo4j graph database using configuration properties and allows for styling the visualization based on nodes, relationships, or specific properties. Note, D3.js is the foundation for Popoto.js, a JavaScript library.

**Neo4j Bloom** ^59^ is a standalone product tool that helps data exploration and visualizes data in the graph, and allows users to navigate and query the data without any query language or programming.

**Grafo** ^60^ is a tool for visually designing knowledge graphs with online, collaborative, real-time editing features. Grafo has reused the existing WebVOWL standard rather than reinventing the wheel.

**WebVOwl** ^61^ is a web framework for interactive ontology visualization. It implements the Visual Notation for OWL Ontologies (VOWL) ^62^ by displaying graphical representations of Web Ontology Language (OWL) elements in a force-directed graph layout that represents the ontology. Exploration of the ontology and customization of the visualization are possible thanks to interaction techniques. The VOWL visualizations are created automatically from JSON files that the ontologies must be translated into. Along with WebVOWL, a Java-based OWL2VOWL converter is included. A comparison of these tools in terms of the several practical issues is presented in Table 4.

**Table 4.** Comparison between knowledge graph visualization options.

	Open source	RDF support	SPARQL Support
Popoto.js	Open source licensed under GNU General Public License v3.0	NA	JavaScript naturally fits for querying a SPARQL endpoint which provides a REST service returning the result in the JSON format
D3.js	open source licensed under BSD license.	NA`	JavaScript naturally fits for querying a SPARQL endpoint which provides a REST service returning the result in the JSON format
Neo4j Bloom	open source licensed under GPLv3.	Yes, via the neosemantics (n10s) plugin.	No, Neo4j has Cypher that covers the need for a structured graph query language.
Grafo	No. offers a Free/Student tier with basic functionality.	supports importing OWL (RDF/XML) and Turtle file formats.	No
WebVOwl	Open source released under the MIT license	visualizations are automatically generated from JSON files into which the ontologies need to be converted	Yes

## 5 Prototype

The ENVRI-KMS development follows an interactive approach, in which prioritized user stories have been analyzed, and technical choices were selected based on the state-of-the-art review done in section 4.4. We use Ontowiki to manage the RDF triples and Open Semantic Search to develop the ENVRI-KMS’s search tool in the current prototype. Several tools were developed for ingesting specific knowledge, e.g., a technology description form for describing the service portfolio, interactive graph visualizer for the search results, and dynamic online data ingestion pipeline. These tools will be described in the following sections.

### 5.1 Knowledge storage

The comparison of existing RDF content management platforms is summarized in Table 2. It was suggested to consider OntoWiki for managing RDF content. The main reasons for this decision were as follows:

(1) Direct operation on RDF triples: Ontowiki can directly operate on a triplestore as the underlying storage layer and provides an API to populate it with RDF.

(2) Integrated User management and statement-level provenance: Ontowiki supports user management with varying permissions and offers a detailed create/update/delete history on the RDF statement level.

(3) Named-graph-based separation of RDF content and administrative data: RDF data ingested via Ontowiki is directly written as-is into the underlying triplestore, while all the administrative statements such as provenance etc., are stored separately.

(4) Plugin-based extensions: Ontowiki offers a framework for developing plugin extensions.

The choice of Ontowiki directly affected the selection of the underlying Triplestore since Ontowiki provides a pre-configured connector to the Openlink Virtuoso data management system, which members of the KB team already had experience with from previous projects. The open-source edition of Openlink Virtuoso ^63^ (Version 7.2.5.1) was therefore deployed for that purpose and configured for Ontowiki (and vice-versa).

### 5.2 Tools for ingesting knowledge

The population of knowledge bases can take different routes. On the one hand, existing collections of information can sometimes be transformed so that they can be "bulk" imported into the ENVRI-KMS, which includes rearrangements and mappings of existing collections of structured information but potentially also the extraction of structured content from unstructured sources such as free text, which is by no means an easy task considering the complexity in the natural language processing/understanding. On the other hand, it is usually possible to manually add ENVRI-KMS’s contents, "fact by fact". However, manual input can be slow, tedious, and error-prone if not supported by dedicated tools. In the context of the ENVRI-KMS, it should be possible to provide content in both ways.

As far as manual data entry is concerned, the system supports the creation of valid RDF data via custom HTML Web forms. They are dynamically created using the RDForms ^64^ Javascript library based on formal JSON descriptions of the underlying data model. This also includes the specification of constrained SPARQL queries for the dynamic retrieval of menu options to maintain consistent RDF relationships between the described entity and related terminology and other entities already stored in the KB.

### 5.3 FAIRness status sharing and gap analysis

A prototype is developed ^5^ to support the discovery of gaps in FAIR principle implementation at the granularity of RI repositories and the discovery of possible technology solutions to address such gaps. By modifying the FAIRness Assessment of a repository of a particular RI—which is functionality natively supported by OntoWiki—for instance, the information on whether or not the repository has machine-readable provenance information, the interface automatically adapts to either include or exclude the corresponding repository under the relevant FAIR principle. By selecting an RI, the user interface presents a summary view for the RI.

### 5.4 Ontowiki as a knowledge management platform

Ontowiki is a suitable RDF data management platform. A test instance is configured ^65^ and slightly customized to use the ENVRI logo and display the ENVRI RSS news feed on the front page. It currently serves as a data gateway for the facts added via forms based on the FAIRness analysis. Ontowiki was found to perform well as RDF "middleware" used to ingest data from the RDF forms. Some issues were discovered regarding the cross-referencing of statements between knowledge bases (named graphs). A workaround published in a newsgroup provided a potential fix for static data but would have to be extended for a continuously growing data collection. A possible solution would be to store information that is expected to change/grow, e.g., the entity descriptions and the user terminology collected from the RDF forms, in a typical named graph and to configure Ontowiki filters for its efficient navigation while storing more static content, such as external ontologies, in separate graphs. While Ontowiki supports flexible navigation and data editing at the RDF statement level, the interface is arguably not appropriate for the vast majority of RI managers or developers. We conducted some experiments with the atmospheric domain, but RIs did not engage with the user interface. This is to be expected since Ontowiki relies on a good understanding of the RDF data model. Moreover, presenting information at the RDF statement’s granularity is typically inadequate for high-level information needs, e.g., discovering FAIR gaps in the data centers of an RI. We thus suggest that Ontowiki can act as an RDF-based middleware that powers high-level user applications and services. A critical aspect of using Ontowiki to manage the generated RDF data will be the question of versioning. While built-in features such as the statement-level provenance in principle allow detailed tracking of changes/revisions of the provided data, a backup strategy using external means should be considered as well. One straightforward step would be to export complete RDF dumps of the provided content in regular intervals and to track their versions in source code repositories such as Github.

### 5.5 Search engine

**Figure 5.**
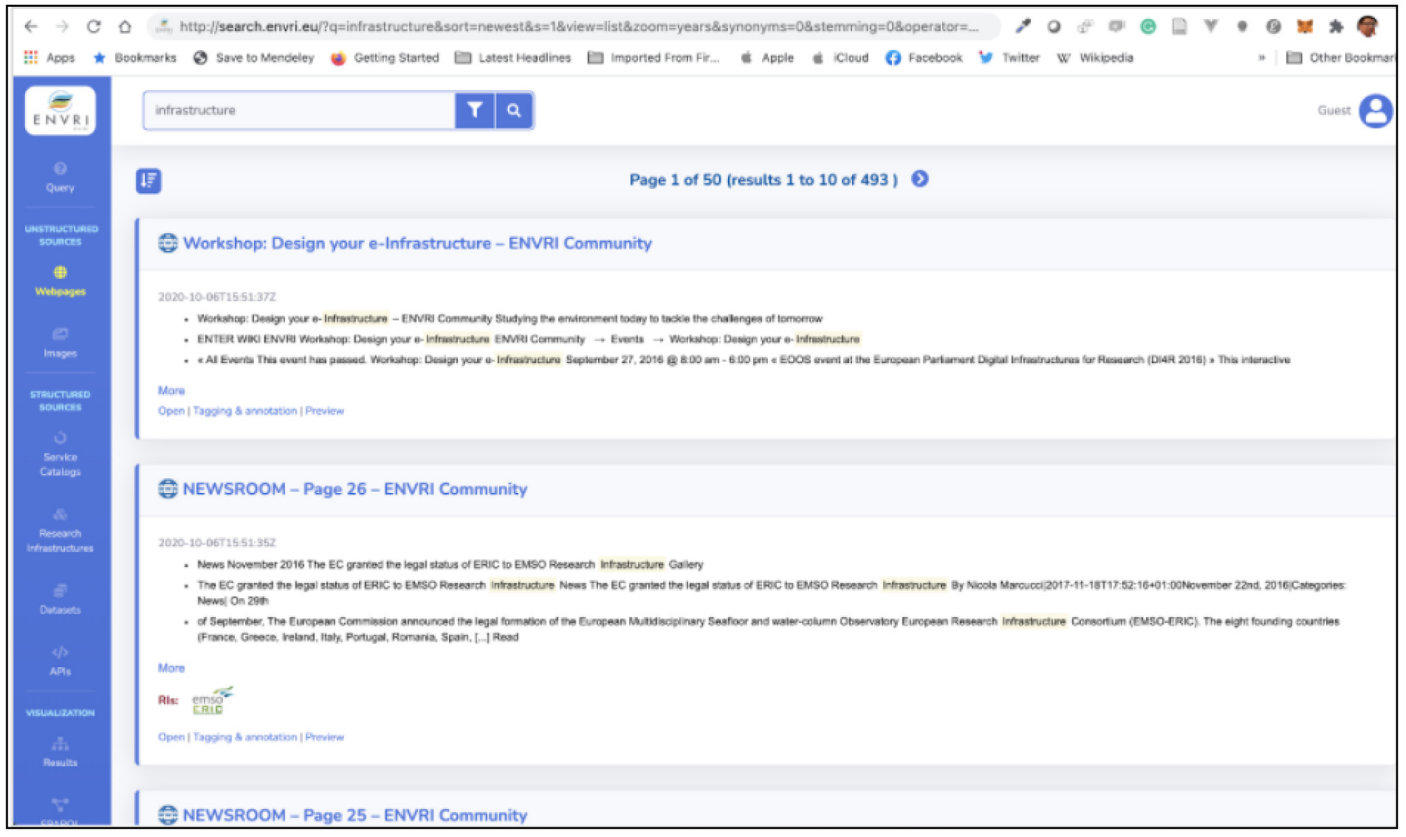
The interface of the ENVRI-KMS.

Though Ontowiki provides navigation functionality over the ENVRI-KMS, it mainly operates at the RDF triple-level, which poses essential technical requirements on users’ expertise. To facilitate the general users to explore the ENVRIKMS easily, we build the ENVRI-KMS Search Engine based on the Open Semantic Search’s fundamental concepts and components. Figure 5 below illustrates the search interface ^6^.

### 5.6 Operational workflow

**Figure 6.**
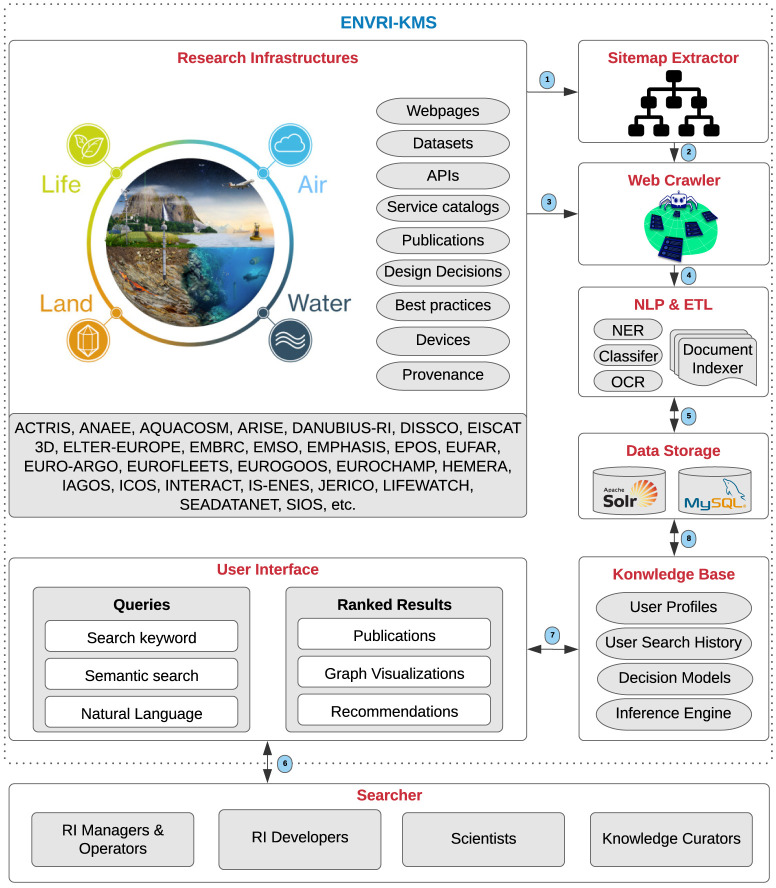
The operational workflow of the ENVRI-KMS.

This section elaborates on the operational workflow of the ENVRI-KMS ^7^ and presents its constituent components (See Figure 6). **Research Infrastructures**, such as ACTRIS and ANAEE, are the primary sources of knowledge that contain knowledge assets, including webpages, datasets, APIs, service catalogs, publications, design decisions, best practices, devices, and data provenance. The **Sitemap Extractor** explores and extracts the site structure (the list of URLs) of the RIs. Then, the **Web Crawler** browses the extracted URLs and by employing **NLP & ETL** (Natural Language Processing and Extract/Transform/Load) techniques, such as Named-Entity Recognition (NER) and Relation Extraction (RE), tries to index documents and classify the extracted knowledge. For instance, in the knowledge extraction process, the NER and RE approaches are used to identify the entities represented in documents and their relations as fundamental knowledge extraction processes. The extracted knowledge is used to build the knowledge graph in the knowledge base of the ENVRI-KMS. **Data Storage** technologies, including Apache Solr and MySQL, are used to store the acquired knowledge systematically. The **Knowledge Base** of the ENVRI-KMS integrates user profiles, user search histories, decision models (e.g., meta-models), and infers solutions (results) based on searchers’ queries. the **User Interface** receives user queries, such as keywords and user stories, and demonstrated the results (e.g., publications, graph visualizations, and recommendations) to the **Searchers the process of extracting useful and hidden information ^14^** (See Section 4.2).

## 6 Analysis

In this subsection, we reflect on each of the proposed research questions based on our observations during the development process, the online survey, and documentation analysis.

### 6.1 Requirements analysis

We revisit the requirements and analyze the gap for the tools or platforms we investigated in terms of the requirements identified in Section 4.1.

**Compatible with Semantic Web technologies.** As the most common type for knowledge storage, representation, reasoning, the support of RDF is the core requirement in designing and developing the ENVRI-KMS. This requirement can include the following specific options, such as RDF import/export, RDF storage, owl import, SPARQL support, etc. The two storage solutions (Apache Jena and Virtuoso) are triplestores dedicated to storing RDF data, thus fully meeting semantic web technology compatibility requirements. Regarding the knowledge management solutions, as the comparison in Table 3 indicates, both Semantic Mediawiki and Ontowiki are RDF compatible.

**Semantic search and query functionality.** An interface for search and discovery of the knowledge base contents should be provided, and this could be the conventional keyword-based search or faceted search. Rather than strict adherence to a single controlled vocabulary or keyword set, a semantic search function is further expected to permit search based on ’similar’ or ’related’ terms.

Though the several Knowledge management systems investigated (like Ontowiki, Semantic Mediawiki) allow users to explore, search and edit the ENVRI-KMS’s content via GUI tools, they still lack easy user experience in terms of the technology required. The original purpose of both Semantic Mediawiki and Ontowiki is a semantic annotation of wiki pages and as a knowledge base editor, respectively.

**Open and flexible knowledge ingestion.** Due to the variance of source types in the ENVRI community, various methods should be supported for knowledge acquisition, like form-based manual RDF ingestion, Questionnaire-based RDF triple generation, existing RDF integration, structured and unstructured information transformation, etc. Specific measures should be considered to facilitate non-technical users straightforwardly adding knowledge.

As shown in Table 2, knowledge management systems, such as Semantic Mediawiki and Ontowiki, support RDF import, facilitating the ingestion of knowledge. However, to prepare RDF triples or transform the information needed into knowledge, some customized tools needed to be designed and implemented considering the diversity of our project’s information sources.

**Provenance and version control of the knowledge.** Considering the typical case where multiple users contribute to the ENVRI-KMS, provenance is of fundamental importance. This primarily refers to tracking individual additions, deletions, and updates and their administration, i.e., approval, rejection, and reversion.

As far as the considered knowledge management platforms are concerned, Ontowiki meets the requirements by providing detailed user management and statement-level provenance for RDF data, allowing tracking and potentially editing individual user contributions to the ENVRI-KMS.

**User-friendly and customizable user interface.** A clear and straightforward user interface is needed to fulfill their objectives, like query, (semantic) search. Advanced services like comparison, recommendation are also needed for interested users. Considering the difference between general public users and professional users, two different user interfaces should be provided.

As already analyzed, although the Knowledge management systems provide a GUI for search and query, their targeted users are knowledge base administrators considering the technology barriers. For general users without much technical knowledge of the SPARQL or triplestores, an easy and straightforward user interface for searching and exploration is expected to increase the user experience.

**Scaling and increasing performance.** A choice between centralized or distributed storage should be considered to tackle the growing size of the ENVRI-KMS. Also should be considered includes the dynamic resource scheduling facing concurrent search/query requests. Other features like collaborative editing are required to enable comments on contributions by other users.

Apache Jena Fuseki does not currently support horizontal scale-up, but there are workaround solutions like coordinating the updates from a staging server and publishing (read-only) to external clients. Based on the comparison, it is clear that no one single solution satisfies all the requirements. The optimal solution should be combining existing options, and other software such as Blazegraph could be a candidate.

### 6.2 Research questions

To answer the first two research questions ( *RQ*_1 _and *RQ*_2_), we have conducted an extensive literature review to build the search space (including RIs, Datasets, etc.) of the ENVRI-KMS and capture knowledge systematically. The current search space ^8^ of the ENVRI-KMS includes all research infrastructures which are mentioned on the ENVRI community knowledge base ^9^. It is essential to highlight the search space is not limited to the initial sets and is growing automatically.

Accordingly, the third research question ( *RQ*_3_) can be addressed based on the natural language processing approach and Open Semantic Search that we have employed in the implementation of the ENVRI-KMS (see Table 3). To answer the fourth research question ( *RQ*_4_), we have evaluated a set of technologies that can be employed to store and retrieve data (see Section 4.4.1, Section 4.4.2, and Section 4.4.3). The last research question ( *RQ*_5_) is one of the key challenges in this research. We plan to build a community around the ENVRI-KMS and ask the stakeholders, including domain experts, practitioners, and researchers, actively assess the search results and recommendations.

The FAIRness of the ENVRI-KMS should be elaborated in order to answer the study ’s main research question. As a result, research assets become **Findable** when adequate metadata characterizes them and a searchable resource efficiently indexes them, allowing them to become recognized and available to potential users. A unique and persistent identifier should also be established so that the data may be referred and mentioned in research communications without ambiguity. The identifier facilitates data discovery and reuse by allowing persistent mapping between data, metadata, and other associated resources. The code or models required to utilise the data, research literature that provides additional insights into the data’s development and interpretation, and other related information are examples of related resources. The ENVRI-KMS indexes research assets and assigns them a unique identifier, allowing them to be shared among RIs.

**Accessibility** means that a human or a machine is given the exact conditions under which research assets can be accessed via metadata. Researchers in research communities can use the ENVRI-KMS to access research assets in accordance with RI policies and regulations.

The ENVRI-KMS search entities are characterized using normative and community-accepted specifications, vocabularies, and standards that define the precise meaning of concepts and qualities represented by the data. **Interoperability** is a crucial aspect of research assets’ value and usefulness. It is not only semantic interoperability that is important, but also technological and legal interoperability. Technical interoperability refers to the research assets being encoded using a standard that can be read by all systems involved.

The FAIR principles highlight the necessity for extensive metadata and documentation that match relevant community standards and give information about provenance in order for research materials to be reusable. The ability of humans and machines to evaluate and select research assets based on provenance information criteria is critical to their reuse.

**Reusability** also necessitates the publication of research assets with a "clear and accessible usage license," which means that the terms under which the assets can be utilized should be transparent to both humans and machines.

Table 5 represents the mapping among the extracted requirements (R01 to R25) based on the responses of the participants to the survey questions (Q1 to Q5) and the research question ( *RQ*_1 _to *RQ*_5_). Additionally, the table shows that more than half of the identified requirements (62% ) are at least partially addressed so that the main components of the ENVRI-KMS are functional.

**Table 5.** The mapping among the extracted requirements (R01 to R25) based on the responses of the participants to the survey questions (Q1 to Q5) and the research question ( *RQ*_1_ to *RQ*_5_). Additionally, the last column shows how far we have addressed the requirements up to now.

Requirements		Survey Questions					Research Questions					Addressed?
		*Q1*	*Q2*	*Q3*	*Q4*	*Q5v*	*RQ1*	*RQ2*	*RQ3*	*RQ4*	*RQ5*	
*R01*	**Completeness of the ENVRI-KMS search space**	X				X	X					Partially
*R02*	**List of the contact persons**	X		X			X					Not yet
*R03*	**FAIRness criteria**	X	X		X						X	Yes
*R04*	**Entitiy types (private, open-source, etc.)**		X				X					Not yet
*R05*	**Recommendations**	X	X		X	X				X		Partially
*R06*	**Q&A forums for technical discussions**		X		X						X	Not yet
*R07*	**Ontologies and semantic search**		X			X				X		Partially
*R08*	**Multilingual queries**		X						X	X		Not yet
*R09*	**Source code and API search**		X							X		Not yet
*R10*	**Search RI website’s contents**		X	X						X		Yes
*R11*	**Standard user interface**			X		X				X		Yes
*R12*	**SPARQL queries**				X					X		Not yet
*R13*	**High performance and availability**				X					X		Partially
*R14*	**APIs to be connected to VREs**				X					X		Partially
*R15*	**Automatic knowledge ingestion**				X	X		X				Yes
*R16*	**Visualization**				X					X		Partially
*R17*	**Image search**				X	X				X		Partially
*R18*	**Assessment tools**		X		X	X					X	Not yet
*R19*	**Manual knowledge ingestion**				X			X				Yes
*R20*	**Updatable knowledge base**					X			X			Yes
*R21*	**Geolocation of the search space entities**		X			X	X					Not yet
*R22*	**Metadata of the search space entities**			X		X	X					Not yet
*R23*	**Continuous integration and continuous** **delivery**					X			X			Yes
*R24*	**Different user categories**					X					X	Partially
*R25*	**Categories & classifications**			X	X		X					Yes

## 7 Discussion

This section summarizes our observations and highlights several lessons learned during the development process of the ENVRI-KMS.

### 7.1 Architecture design

Software architecture deals with the base structure, subsystems, and interactions among these subsystems, so it is critical to the success or failure of any software system ^67^. Software architecting can be thought of as a decision-making process in which software architects consider a collection of possible solutions for solving a system design problem and choose the one that is evaluated as the optimal ^68^. Software architecture decisions are design decisions that meet both functional and quality requirements in a system.

Design decisions are concerned with the system’s application domain, architectural patterns employed in the system, Commercial off-the-shelf components, other infrastructure selections, and other aspects needed to satisfy all requirements ^69^. According to Avgeriou *et al*. ^70^, failing to make architectural design decisions during software development has well-known implications, such as costly system evolution, weak stakeholder communication, restricted reusability of architectural assets, and poor traceability between specifications and implementation.

Each architectural design decision is made with a design rationale ^71^, which represents the knowledge that provides the answers to questions about the design decision or the process followed to make that decision. In literature, various researchers such as Babar *et al*. ^72^, and Avgeriou *et al*. ^70^ have highlighted the necessity of document design rationale to maintain and evolve software products and avoid violating rules of design decisions underpinning the original architecture. Design rationale is an essential part of an architecture description according to the IEEE 1471 recommended practices ^73^.

Architectural knowledge needs to be documented and codified in some way so that it can be searched and retrieved at different times ^74^. Software systems’ high-level architecture and behaviors are described by architectural patterns. It solves a specific reoccurring challenge in software architecture design within a particular context ^75^. Software product audits should not be viewed as as isolated project, according to 76. Individual audits, on the other hand, have an impact on each other, even if they target unrelated software products. Lessons learned in one project, for example, may be applicable to another.

In addition, the applicability of specific quality criteria is not restricted to a single project. Some general quality criteria may apply to almost all software products, and similar projects may utilize comparable quality criteria. User authentication will always be required in high-security knowledge management systems, for example. As a result, making well-informed design decisions is an expertise. Novice software engineers, no of how good they are, would not have a large set of known and experienced scenarios, design challenges, or practical solutions to pattern match current problems ^77^.

### 7.2 Implementation

Software engineers have a broad knowledge of software development technologies, and they apply software engineering principles to develop software products. By employing such engineering principles in the software development lifecycle, from requirements elicitation to software implementation and then deployment, they can build customized software products for individual stakeholders.

The demand for highly skilled and qualified software engineers seems to have no end. This demand is growing in a changing economic landscape and fueled by the necessity of software development technologies. On the one hand, billions of dollars are spent annually on software products ^78^ that are produced and maintained by software engineers. On the other hand, business processes are introduced and managed by stakeholders and top-level managers who principally understand businesses ^79^.

Software development is not an independent activity: it typically requires interactions with stakeholders, which necessitate a level of agreement in the description of the technical phases of development. Moreover, software products are getting more complicated, so that they need to be discussed at different abstraction levels depending on the technical background of the involved domain experts, phase of the development process, and business objectives ^80^. Modeling is a handy tool for addressing such issues in software production as it simplifies the technical complexities and improves system understanding through visual analysis.

Judging the suitability of a set of technologies, such as programming languages, for developing a knowledge base system is a non-trivial task. For instance, a purely functional language like Haskell is the best fit for writing parallel programs that can, in principle, efficiently exploit huge parallel machines working on large data sets ^81^. However, while developing a dynamic website, a software engineer might consider ASP.net as the best alternative, and others might prefer using PHP or a similar scripting language. It is interesting to highlight that successful projects have been built with both: StackOverflow is built in ASP.net, whereas Wikipedia is built in PHP. Furthermore, a software engineer might prefer particular criteria, such as scalability in enterprise applications, whereas other criteria, such as technology maturity level, might have lower priorities.

Acquiring and expanding knowledge about programming languages is a highly complex process, as significant numbers of criteria and alternatives exist in the market ^78^. Various factors need to be taken into account, of which not all are obvious. Simultaneously, the choice of technologies can have repercussions on the implementation cost, quality of the result, and maintenance cost of the application ^82^.

## 8 Conclusion and future work

The development and operation of the ENVRI-KMS will be continuous. It will grow during the project while the development results and knowledge accumulate. The ENVRI-KMS development and operation depend on the development effort from the ENVRI subdomains and research infrastructures. The ENVRI-KMS should play a role in supporting developers from RIs to share best practices and find existing solutions, but the ENVRI community provides valuable input to the ENVRI-KMS and keeps it alive.

Currently, the ENVRI-KMS team closely interacts with the other subdomain developers (via workshops, meetings, and workgroups organized by subdomains). Through members, there is valuable input of the catalog of services, authentication and authorization, persistent identifier, triple store, license and usage tracking, and ENVRI-HUB.

The ENVRI-KMS team also closely interacts with semantic search workgroups in subdomains, e.g., a semantic search use case in ACTRIS ^10^ reported in the Semantic Search Working Group Final Report []FinalReport.

The ENVRI-KMS will continue in the rest of the ENVRI-FAIR project. In the next phase, the development effort will mainly focus on the following aspects: (1) Continuous content ingestion and curation. The ENVRI-KMS team will improve the knowledge ingestion tool and continuously ingest the description (metadata) of high-quality results from the ENVRI community (e.g., sub-domain or RI developers), including development results (e.g., best practices, software technologies, recommendations, updated FAIRness assessment possibly generated by new tools) in the ENVRI-KMS, and make those descriptions FAIR for the community.

(2) Continuous improvement of the ENVRI-KMS based on the feedback is received from the community. Extra features, e.g., for ENVRI-KMS discovery and recommendation, will be further explored.

(3) The development and operation of the ENVRI-KMS will also follow the software engineering DevOps practices. The continuous testing, integration, and deployment pipeline will be established.

(4) We will also extend the content maintenance to community specialists. In this way, we hope the community will play a key role in the ENVRI-KMS.

## Data availability

### Underlying data

Mendeley Data: ENVRI-KMS. https://doi.org/10.17632/ntxypfsvds.1 ^42^

This project contains the following underlying data:

- Knowledgebase-discussion.xlsx (Raw survey outcome data)

### Extended data

Mendeley Data: ENVRI-KMS. https://doi.org/10.17632/ntxypfsvds.1 ^42^

This project contains the following extended data:

- ENVRI-KMS (1).pdf (Summary of data analysis)
- Knowledgebase-discussion.pdf (Visualized survey outcomes)

Data are available under the terms of the Creative Commons Attribution 4.0 International license (CC-BY 4.0).

## Software availability

Software available from: SciCrunch: ENVRI-KMS,RRID:SCR_021235

Source code available from: https://github.com/SiamakFarshidi/solr-php-ui.git

Archived source code at time of publication: http://doi.org/10.5281/zenodo.4882766 ^41^

License: https://opensource.org/licenses/Apache-2.0

## Footnotes

^1^FAIR data are data that meet principles of findability, accessibility, interoperability, and reusability.

^2^ENVRI-HUB is a one-stop-shop for access to environmental data and services provided by the contributing research infrastructures.

^3^We have published the responses of the domain experts who participated in the survey besides the data analysis phases on Mendeley Data ^46^.

^4^PHP (Hypertext Preprocessor) is an open source general-purpose scripting language that is especially suited for web development and can be embedded into HyperText Markup Language (HTML).

^5^The prototype can be accessed at the following address: https://envri-fair.github.io/knowledge-base-ui/

^6^The search instance is available through the following link: https://search.envri.eu

^7^In order to implement the ENVRI-KMS, we have employed the proposed design decisions and solutions that Open Semantic Search ^66^ and WebVOWL ^62^ as two open source projects have been offered.

^8^ https://search.envri.eu/

^9^ https://envri.eu/research-infrastructures/

^10^ https://github.com/xiaofengleo/actris
