# Knowledge sharing and discovery across heterogeneous research infrastructures

**DOI:** 10.12688/openreseurope.13677.2

**Published:** 2021-11-22

**Authors:** Siamak Farshidi, Xiaofeng Liao, Na Li, Doron Goldfarb, Barbara Magagna, Markus Stocker, Keith Jeffery, Peter Thijsse, Christian Pichot, Andreas Petzold, Zhiming Zhao

**Affiliations:** 1 MultiScale Networked Systems (MNS), University of Amsterdam, Amsterdam, Netherlands, 1098 XK, The Netherlands; 2 Environment Agency Austria, Vienna, Austria; 3 TIB – Leibniz Information Centre for Science and Technology, Hannover, Germany; 4 British Geological Survey, London, UK; 5 MARiene Informatie Service, Nootdorp, The Netherlands; 6 French National Institute for Agriculture, Food, and Environment, Paris, France; 7 Forschungszentrum Juelich GmbH, Jülich, Germany

**Keywords:** Knowledge base, knowledge management, search engine, research infrastructure, software development lifecycle

## Abstract

Research infrastructures play an increasingly essential role in scientific research. They provide rich data sources for scientists, such as services and software packages, via catalog and virtual research environments. However, such research infrastructures are typically domain-specific and often not connected. Accordingly, researchers and practitioners face fundamental challenges introduced by fragmented knowledge from heterogeneous, autonomous sources with complicated and uncertain relations in particular research domains. Additionally, the exponential growth rate of knowledge in a specific domain surpasses human experts’ ability to formalize and capture tacit and explicit knowledge efficiently. Thus, a knowledge management system is required to discover knowledge effectively, automate the knowledge acquisition based on artificial intelligence approaches, integrate the captured knowledge, and deliver consistent knowledge to agents, research communities, and end-users. In this study, we present the development process of a knowledge management system for ENVironmental Research Infrastructures, which are crucial pillars for environmental scientists in their quest for understanding and interpreting the complex Earth System. Furthermore, we report the challenges we have faced and discuss the lessons learned during the development process.

## 1 Introduction

Contemporary societies are faced with a new challenge for the ’globe’ – the changing of the world’s climate ^1^. Climate change is unpredictable in its form and scope and is long-term rather than immediate in its impacts and remedies. Any practical solutions lie beyond any act of national will, requiring the international collaboration of unprecedented dimension and complexity. While an effective solution to address the challenge would play out over several decades, it is required to be shaped and put in place over the next few years ^2^.

Climate change has been identified as a major environmental problem for humanity by the United Nations and the European Union. Research is expected on potential scenarios on climate change that will drastically affect natural ecosystems, plants, habitat, and animals, contributing to speedup in biodiversity loss in some areas. The impacts would have knock-on effects for many communities and sectors that rely on natural resources, including agriculture, fisheries, fuels, tourism, and water. Additionally, the ocean plays a central role in regulating the Earth’s climate ^3^.

 Assessments of climate change and their association with the driving forces must be based on trustworthy and well-documented observations. This is a difficult task due to the many interactions that exist between the atmosphere, soil, and hydrosphere. The resulting impacts on ecosystems all need particular and focused, high-quality long-term observations. This forces us to have better observations and data on these essential preconditions to inform decision-makers better to take the measures necessary to maintain a thriving society ^4^.

Research Infrastructures (RIs) are vital for providing the required information to support science and fact-based policy development. Research infrastructures, including advanced computing and storage infrastructure, in environmental science, are essential requirements for scientists in this domain to understand and analyze the sophisticated earth system ^5^. Interdisciplinary research communities and research infrastructures collaborate with the neighboring disciplines, namely atmosphere, biosphere, hydrosphere, and geosphere. Internal cooperation across different realms resulted in the formation of distinct research traditions, skills, and cultures. The interconnected essence of the earth system, on the other hand, requires the scientific community to transcend well-established divisions between disciplines and domains and work toward a common understanding of the world as a whole ^6^.

The data from the ICOS ^1^ Research Infrastructure, for example, aids climate science by informing scientists and the general public on natural and human-caused greenhouse gas emissions and uptake from the ocean, land ecosystems, and atmosphere. It gives access to high-quality data processed by the Thematic Centers as raw, near real-time, and final quality-controlled data and supplemented with elaborated (model) data and analyses, almost always licensed under a CC4BY ^2^ license. The IAGOS ^3^ research infrastructure provides atmospheric composition information, including greenhouse gas observations from commercial aircraft. IAGOS data are used by researchers worldwide for process studies, trend analysis, validation of climate and air quality models, and spaceborne data retrievals validation. Aerosols and their precursors are monitored by the ACTRIS ^4^ research infrastructure. Aerosols have a significant impact on the Earth’s radiation balance, and consequently, the climate. Their levels are inextricably linked to human activity and emissions. Such RIs are part of a more significant worldwide effort to advance science-based, high-quality observations that will help people in making better decisions. As a result, the data and procedures are based on international, typically community-based standards.

Typically, RIs are domain-specific and are not connected, so that interoperability can be a critical issue for scientists involved in interdisciplinary research projects. Moreover, researchers/developers are not knowledgeable in all domains, so a knowledge management system is required to capture cross-domain environmental knowledge automatically and enable researchers to access data, software tools, and services from different sources and integrate them into cohesive experimental investigations with well-defined, replicable workflows for processing data and tracking results’ provenance. Accordingly, a knowledge management system is required for research communities that (1) discover cross-domain knowledge and capture them automatically, (2) answer any domain question without any limitation to its current search space, (3) deal with noisy sets of retrieved documents, likely consisting of many irrelevant documents and semantically and syntactically ill-formed documents, (4) have an advanced search engine to interpret and reformulate queries by information retrieval algorithms, (5) return a set of recommended solutions (answers) based on the retrieved documents, and (6) visualize its outcomes to facilitate the data analysis for research communities. This paper introduces a novel Knowledge management system, called ENVRI-KMS, to meet the ENVRI research community’s requirements and make the research assets Findable, Accessible, Interoperable, and Reusable (FAIR ^10^) for the community.

The rest of this study is structured as follows: Section 2 introduces knowledge discovery and sharing challenges, formulates the design research questions, and elaborates on the research methods that have been employed to capture knowledge regarding the ENVRI-KMS.

Section 3 outlines the development process of the ENVRI-KMS. Section 3.1 explains the online survey that we conducted to collect requirements of the ENVRI-KMS. Section 3.2 shows the use case scenarios that we identified based on the survey. Section 3.3 introduces the design decisions that we made to design the ENVRI-KMS architecture. Section 4 elaborates on the selected technologies that we employed to develop the ENVRI-KMS and demonstrates part of the current implementation. Section 5 analyzes the requirements and maps them to the survey questions and design research questions based on the participants’ responses. Section 6 highlights the challenges and lessons learned during the development process of the ENVRI-KMS. Section 7 positions the proposed approach in this study among the other knowledge management approaches in the literature. Finally, Section 8 summarizes the proposed approach, defends its novelty, and offers directions for future studies.

## 2 Challenges regarding knowledge sharing and discovery

In this paper, we present a novel knowledge management system, called ENVRI-KMS, to meet the ENVRI research community requirements and make the research assets Findable, Accessible, Interoperable, and Reusable (FAIR ^11^) for the community. The ENVRI-KMS is a Knowledge-as-a-Service (KaaS) for ENVRI-FAIR research communities to document the development and operation processes of RIs and support them with their engineering and design decisions. In general, the ENVRI-KMS should (1) ingest technical results from ENVRIplus, FAIR assessment ^5^, the key sub-domains, and other tasks using a formal language for knowledge representation and proven semantic technologies; (2) provide services and tools to enable RI developers and data managers to browse, search, retrieve and compare RI technical statuses and technical solutions to development problems via available content; (3) provide content management tools for specialists in the ENVRI community to ingest new knowledge and control the quality of content; (4) also provide interfaces to other existing semantic resources, e.g., the service catalog of a future ENVRI-HUB ^6^, to enhance knowledge discovery and cross-RI search, between knowledge services and the online presence of ENVRI resources.

A significant number of advanced research infrastructures, such as ICOS ^7^ and IAGOS ^8^, are available to facilitate the access of researchers to research assets (e.g., data products, best practices, data service design decisions, software tools, and services). Such research assets are scattered among a wide range of heterogeneous knowledge resources ^5^. Furthermore, operational policies of different domains typically restrict interoperability and accessibility of multidisciplinary research projects. Additionally, technical reports about architectural design, service interfaces, selections of metadata standards, controlled vocabularies, and ontologies are not shared effectively. Accordingly, the main design research question in this study is *"How to enable a domain-specific research community with their asset discovery challenges based on the FAIR principles?"*

As knowledge is scattered in a wide range of literature, forums, documentation, and tacit knowledge of domain experts, the following design research questions should be addressed to capture knowledge systematically: *RQ*1: Which sources of knowledge should be employed to build the search space of the the ENVRI-KMS? *RQ*2: How to capture knowledge regarding RI’s research assets systematically? *RQ*3: How to keep the knowledge base of the ENVRI-KMS always up to date? *RQ*4: How to store and retrieve acquired knowledge when it is needed by the ENVRI-FAIR communities? *RQ*5: How to evaluate the recommended solutions of the ENVRI-KMS?

This study employs a mixed research method based on design science research, surveys, and documentation analysis to capture knowledge regarding knowledge management systems and answer the design research questions. The research approach for creating the proposed knowledge management system, called ENVRI-KMS, is Design Science, which addresses research by building and evaluating artifacts to meet identified business needs ^11^ in an iterative process ^12^. Furthermore, we designed a survey form and asked several of our colleagues to critique it. We conducted an online survey in the context of 26 research infrastructures to collect their functional requirements and quality concerns. In total, 35 domain experts participated in the research to assist us with the ENVRI-KMS development life cycle and the requirement analysis phase. Moreover, to develop the ENVRI-KMS, we reviewed webpages, whitepapers, scientific articles, fact sheets, technical reports, product wikis, product forums, product videos, and webinars to collect data. A structured coding procedure is employed to extract knowledge from the selected sources of knowledge.

Knowledge management systems employ problem-solving techniques, and knowledge discovery approaches to answer particular questions ^13, 14^. Knowledge discovery is the process of extracting useful and hidden information ^15^. A variety of Knowledge management systems have been introduced in literature ^16– 18^. Most of the existing knowledge management systems in the literature bound to a limited search space and optimized to address questions in a particular context. Each question-answer-context tuple is well-formed, standardized, and generated rising from the context in which the question and answer were extracted.

## 3 ENVRI knowledge management system

The ENVRI-KMS ^19^ is a cluster-level knowledge base that allows different ENVRI users, such as RI developers and data managers, to effectively share their technical practices, identify common data and service requirements and design patterns, and facilitate the search and analysis of existing RI solutions for environmental RI interoperability challenges.

### 3.1 Requirement analysis

We organized a webinar for conducting an online survey in the context of 26 research infrastructures, currently active in the ENVRI-FAIR project ^20^, to collect their functional requirements and quality concerns ^21^. Thirty-five domain experts participated in the research to assist us with the ENVRI-KMS development life cycle and the requirement analysis phase. The domain experts were selected according to their expertise and years of experience. On average, the participants had more than ten years of experience within their expertise. They were totally aware of potential challenges that researchers in their community and field might face while performing their daily tasks. Firstly, we introduced the potential functionality of the ENVRI-KMS and presented some of its applications according to the literature study and internal meetings that we have conducted with a limited number of domain experts. Then, we used an online survey tool, called Mentimeter ^22^, to distribute a virtual questionnaire including the following questions: (Q1) What information will you typically search from the ENVRI community? (Q2) What will be the typical queries you would ask the ENVRI-KMS? (Q3) How do you currently search for information from the ENVRI community? (Q4) Which knowledge management system functionality do you feel is most beneficial for you? (Q5) What function do you expect from the next version of the ENVRI-KMS?

Next, we have collected all responses and prioritized them based on analyzing the frequencies of similar statements ^7^.

Table 1 shows the requirements that we have extracted from the experts’ responses. The color-coding indicates the importance of each requirement according to the number of responses that signified it.

**Table 1.** shows the requirements that we have extracted from the experts’ responses. The color-coding indicates the importance of each requirement according to the number of responses that signified it.

ID	Requirements	doamin experts
***R01***	*The ENVRI-KMS should include all prospective RIs, datasets, repositories, best practices, service catalogs, and design* *decisions in its search space.*	54.29%
***R02***	*Contact lists of people in charge of specific tasks (authors, researchers, developers, etc.) must be provided by the ENVRI-* *KMS.*	54.29%
***R03***	*The ENVRI-KMS is required to employ a set of assessment criteria, such as FAIRness criteria, to evaluate search space* *entities.*	85.71%
***R04***	*Private, public, open-source, or premium search space entities should be indicated in the ENVRI-KMS.*	37.14%
***R05***	*Documentation, technical solutions, configurations, and compatible combinations should all be recommended by the* *ENVRI-KMS.*	34.29%
***R06***	*The ENVRI-KMS should provide technical discussions through Q&A forums and invite domain experts to participate.*	45.71%
***R07***	*Ontologies and semantic search should be supported by the ENVRI-KMS.*	60.00%
***R08***	*Multilingual inquiries should be supported by the ENVRI-KMS.*	8.57%
***R09***	*The ENVRI-KMS should be able to search for source code and provide suitable solutions to technical issues.*	14.29%
***R10***	*The contents of the RI websites have to be searchable through ENVRI-KMS (similar to what Google search engine does.)*	60.00%
***R11***	*The user interface (UX/UI) of the ENVRI-KMS should be similar to typical search engines.*	57.14%
***R12***	*The ENVRI-KMS should be able to connect to endpoints and support SPARQL queries.*	28.57%
***R13***	*High performance and availability have to be two essential quality attributes of the ENVRI-KMS.*	68.57%
***R14***	*APIs for connecting to virtual research environments (ENVRI-HUB) should be available through the ENVRI-KMS.*	34.29%
***R15***	*Automated knowledge ingestion should be possible with the ENVRI-KMS.*	28.57%
***R16***	*The outcomes and contents of the ENVRI-KMS should be visualized.*	48.57%
***R17***	*The ENVRI-KMS should be able to search numerous image categories (plots, etc.) and support image search.*	42.86%
***R18***	*Domain experts must be able to analyze the contents of the ENVRI-KMS using assessment techniques.*	48.57%
***R19***	*The ENVRI-KMS should support manual knowledge ingestion.*	11.43%
***R20***	*The knowledge base of the ENVRI-KMS should always be kept up-to-date.*	31.43%
***R21***	*One of the search criteria in the ENVRI-KMS should be the geolocation of datasets.*	48.57%
***R22***	*The metadata of the search space entities, such as datasets and APIs, should be available through the ENVRI-KMS.*	20.00%
***R23***	*Continuous integration and continuous delivery (CI/CD) should be supported by the ENVRI-KMS.*	28.57%
***R24***	*Different user groups, such as researchers, knowledge curators, developers, and high-level managers, should receive* *suggestions from the ENVRI-KMS.*	17.14%
***R25***	*The ENVRI-KMS should categorize and classifies its knowledge base contents.*	37.14%

The initial user stories for the ENVRI-KMS mainly focus on the data manager, RI service, or Virtual Research Environment (VRE) ^23, 24^ developers, e.g., for enabling a developer to check the existence or details of data management solutions from different RIs. Accordingly, the following key technical requirements have been identified to design and implement the ENVRI-KMS:

**Compatible with semantic web technologies.** As the most common type for knowledge storage, representation, reasoning, the support of Resource Description Framework (RDF) is the core requirement in the design and development of the ENVRI-KMS. This requirement can include the following specific options: RDF import/export, RDF storage, owl import, SPARQL, and GeoSPARQL support. It is acknowledged that while providing many advantages, especially in the context of integrating and operating on heterogeneous knowledge sources and of linking to existing external resources, RDF, but also the overall concept of operating on a non-monolithic set of data collections, comes with specific limitations as well, such as lack of support for referential integrity. Nevertheless, it is assumed that the ENVRI-KMS content’s nature is rather non-volatile, shifting this aspect into the background.

**Semantic search and query functionality.** An interface for searching and discovering ENVRI-KMS content should be provided; this could be the conventional keyword-based search or faceted search. A semantic search function is further expected to permit search based on ’similar’ or ’related’ terms across multiple ontologies/controlled vocabularies rather than strict adherence to a single controlled vocabulary or keyword set ^25^.

**Open and flexible knowledge ingestion.** Due to the variance of source types in the ENVRI community, various methods should be supported for knowledge acquisition, like form-based manual RDF ingestion, Questionnaire-based RDF triple generation, existing RDF integration, structured and unstructured information transformation, etc. Specific measures should be considered to facilitate non-technical users straightforwardly adding knowledge.

**Provenance and version control of the knowledge.** Considering the typical case where multiple users contribute to the ENVRI-KMS, provenance is of fundamental importance for monitoring and tracking issues, for example, enabling the third party to reproduce the scientific workflow for an authority to audit the whole process. This primarily refers to tracking individual additions, deletions, and updates and their administration, i.e., approval, rejection, and reversion.

**User-friendly and customizable user interface.** A clear and straightforward user interface is needed to fulfill their objectives, like query, semantic search. Different user interfaces should be offered to meet the requirements of the general public and professional users.

**Scaling and increasing performance.** A choice between centralized or distributed storage should be considered to tackle the growing size of the ENVRI-KMS. Also should be considered includes the dynamic resource scheduling facing concurrent search/query requests. Other features like collaborative editing are required to enable comments on contributions by other users.

**API interface.** An application programming interface (API) abstraction layer can help make knowledge accessible through applications to facilitate knowledge via APIs.

Among such technical requirements, the ENVRI-KMS should play a key role in the ENVRI communities to develop FAIR data services and share their best practices.

### 3.2 Use case scenarios

**Figure 1.**
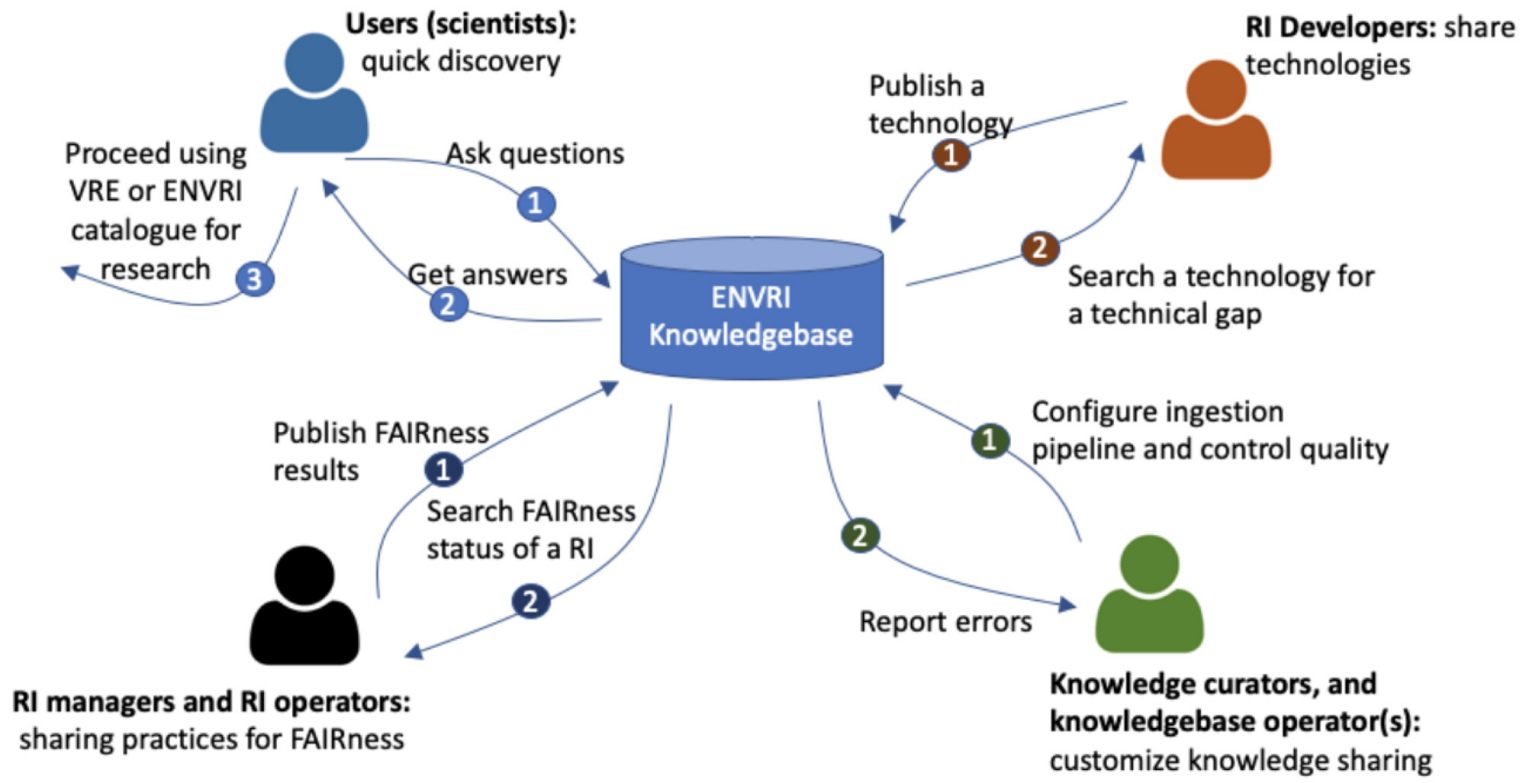
Shows an enterprise view of the ENVRI-KMS. The enterprise view highlights the key stakeholders (namely communities in the Open Distributed Processing (ODP) term) and their interaction scenarios with the ENVRI-KMS. The numbered circles indicate the possible orders of the interactions.

Based on the survey we conducted (see section 3.1), we identified the following four types of users (see Figure 1) of the ENVRI-KMS:

(1) **End users** may use the ENVRI-KMS to find answers to their general questions about available sources of data, services, and tools, and to use the discovered information to perform further research activities using the other tools like Virtual Research Environments or services like the RI catalogs of data or services.

(2) **RI managers** or operators may use the ENVRI-KMS to check the status of the FAIRness of specific repositories or update the state of their RIs. The update process often needs the output of other third-party tools, e.g, FAIRness assessment tools.

(3) **RI developers** may use the ENVRI-KMS to check the existing technologies, e.g., those development results in the ENVRI portfolio or the demonstrators prepared for some known FAIRness gaps. They can also publish or update the technical descriptions using ENVRI-KMS components, such as an online description form.

(4) **Knowledge curator and knowledge base operators** may use the ENVRI-KMS to ingest content from new sources and respond to the possible errors that occurred during the ingestion or the operation.

### 3.3 Conceptual architecture

**Figure 2.**
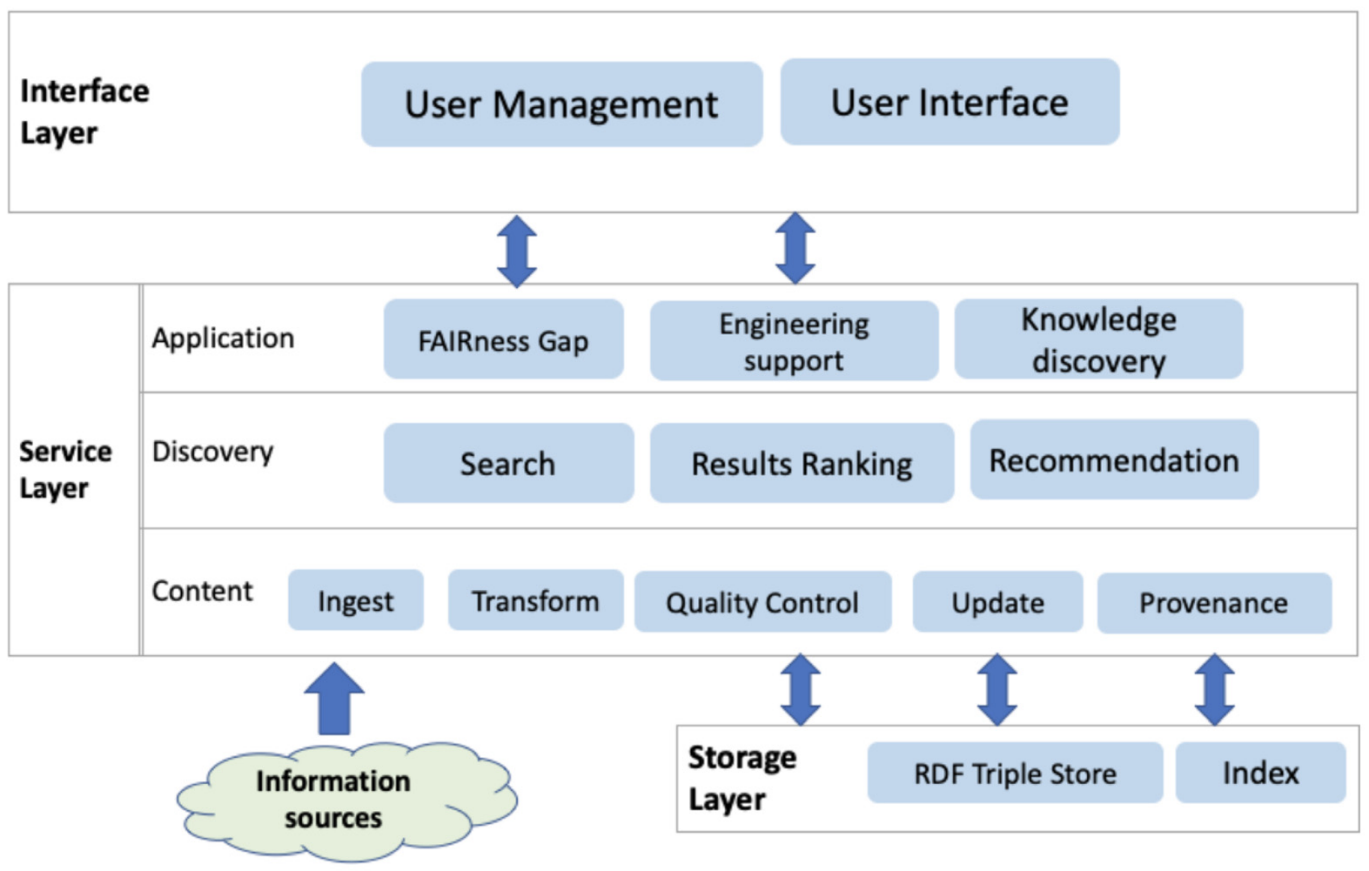
Shows the layered architecture of the ENVRI-KMS that is designed based on the Open Distributed Processing framework.

Based on the use case scenarios (see Figure 1), we design the key components of the ENVRI-KMS from the conceptual point of view. Note, the architecture is designed based on the Open Distributed Processing (ODP) framework ^26– 29^. Figure 2 shows the key components via three layers:

The **interface layer** atop contains components dealing with user-related activities. The ENVRI-KMS will be an open system for community users; the user management component is not for acquiring and processing users’ personal information but more for providing customized user support based on their interaction or contexts. A user can log in to the system using an open identity provider. The User Interface (UI) components are the application parts that allow users to interact with it. It can be formatted and rendered into various presentations to address different users’ requirements. Additionally, it validates and collects required data from users.

The **service layer** abstracts the functionality that the ENVRI-KMS offers; it can be roughly split into three sub-layers, namely:

(1)   The Application sub-layer provides customized application logic (e.g., FAIRness Gap Analysis, Engineering support, or discovery knowledge from ENVRI community) based on the data passed from the underlying discovery sub-layer those results up to the User Interface Component.

(2)   The Discovery sub-layer provides the functionality for searching the ENVRI-KMS, ranking the results, and recommending relevant content.

(3)   The Content sub-layer provides functionality for managing the content in the ENVRI-KMS, typically in a pipeline covering: ingesting information, the transformation from information to knowledge, quality control of the knowledge generation, CRUD (Create, Read, Update, Delete) of the ENVRI-KMS content, and the provenance of these activities.

**Figure 3.**
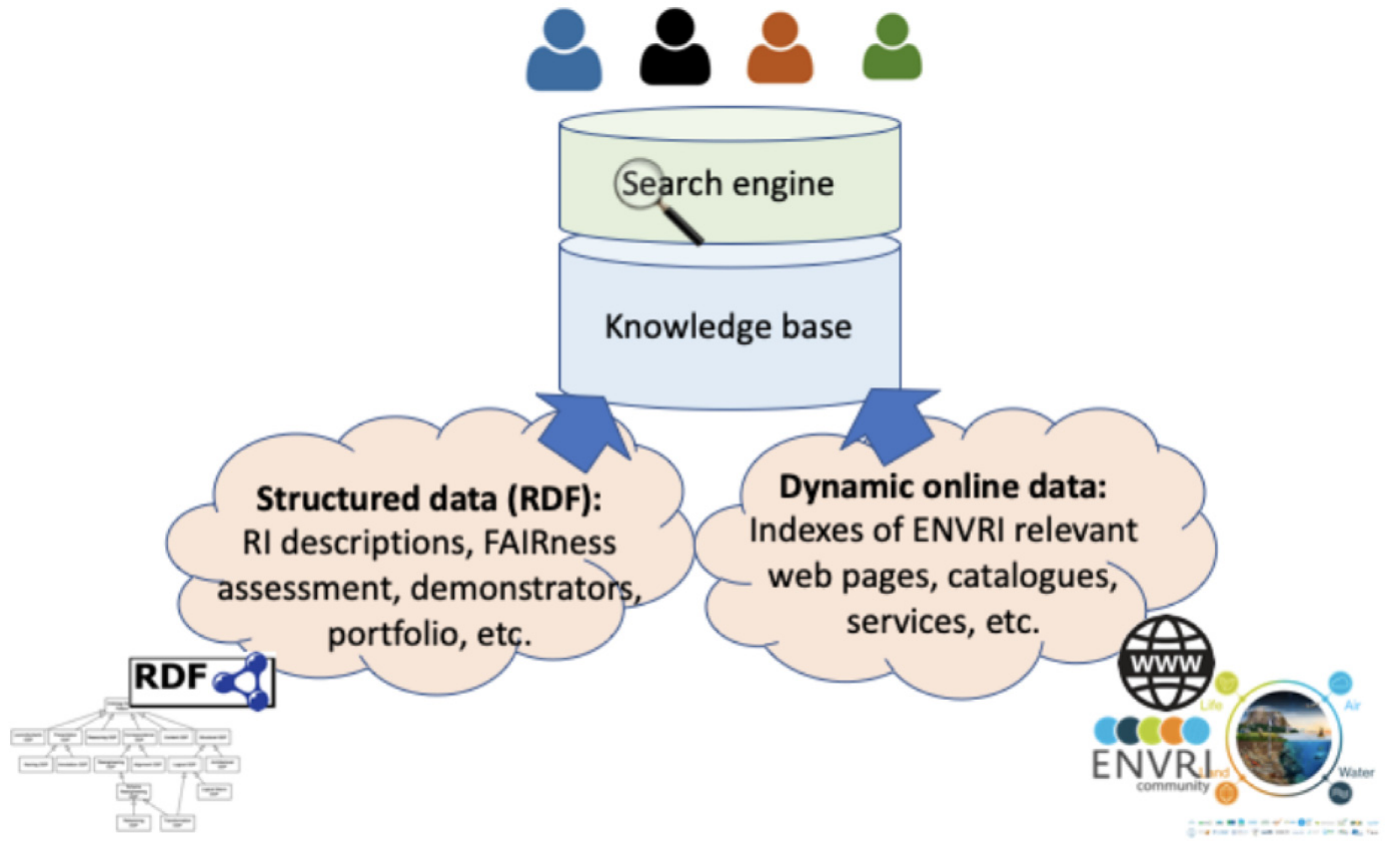
Illustrates the ENVRI-KMS content components. The ENVRI-KMS can be used by end users to search different contents.

The **storage layer** at the bottom is responsible for data storing and access. The data storage options needed in this project include RDF Triple Store and Inverted Index. Currently, information collected in the ENVRI-KMS consists of two main parts, as illustrated in Figure 3. The structured data in the ENVRI-KMS is based on RDF and mainly includes: (1) OIL-e (ontology of the ENVRI Reference Model) based ENVRI RI description, (2) description of the service portfolio from the previous project, and the possibly new ones in ENVRI-FAIR, (3) FAIRness principles and the results of assessing the ENVRI research infrastructures, and (4) demonstrators for tackling the known gaps, e.g., those being identified during the FAIRness assessment.

**Figure 4.**
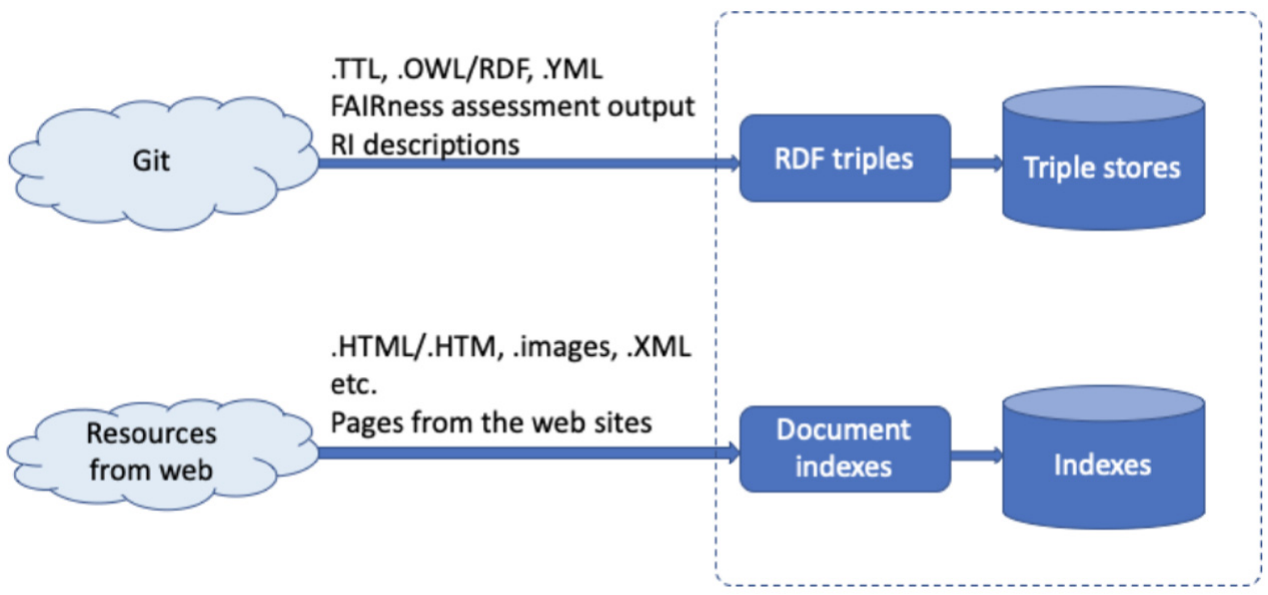
Shows the basic information flow of the knowledge ingestion.

The versions of the structure data currently can be managed via version control systems. Currently, GitHub is used. The dynamic data in the ENVRI-KMS will be ingested from different online sources of the ENVRI communities. Figure 4 depicts the necessary information flow of the knowledge ingestion.

(1)   A significant amount of relevant information is represented in human-readable form, residing in Wikis, other content management systems, or even static web-pages, in the "offline" text found in various documents such as books, project deliverables, or scientific publications. In the ENVRI-FAIR context, the research infrastructure websites are an excellent resource of related information, including news/events, background knowledge, etc. Similar to ENVRI, ENVRI-FAIR, the community websites also contain lots of related information, like news/events, community introduction, community landscape, project information, progress, etc. These information sources have different formats, such as a webpage, word document, and pdf file.

(2)   Another approach to populate the ENVRI-KMS would be to process such free-text information to extract structured, machine-readable information. Named entity recognition would represent the first step in this regard, while the application of more complicated Natural Language Processing operations could be a valuable field of research in its own right.

(3)   Information from the available catalogs of data and services. It should be clear that the indexes generated from those sources will not aim to replicate the entire catalogs but provide a quick searching capability for community users. For some RI, such information will be already managed in RDF format and accessible from triplestores.

## 4 Prototype

The ENVRI-KMS development follows an interactive approach, in which the requirements based on the experts’ responses (see Section 3.1) have been analyzed, and technical choices have been selected according to the state-of-the-art review published in 30. We use Ontowiki to manage the RDF triples and Open Semantic Search to develop the ENVRI-KMS’s search engine in the current prototype. Several tools were developed for ingesting specific knowledge, e.g., a technology description form for describing the service portfolio, interactive graph visualizer for the search results, and dynamic online data ingestion pipeline. These tools will be described in the following sections.

### 4.1 Knowledge storage

The comparison of existing RDF content management platforms is summarized in 30. Note, we have selected OntoWiki for managing RDF content. The main reasons for this decision were as follows:

(1)   Direct operation on RDF triples: Ontowiki can directly operate on a triplestore as the underlying storage layer and provides an API to populate it with RDF.

(2)   Integrated User management and statement-level provenance: Ontowiki supports user management with varying permissions and offers a detailed create/update/delete history on the RDF statement level.

(3)   Named-graph-based separation of RDF content and administrative data: RDF data ingested via Ontowiki is directly written as-is into the underlying triplestore, while all the administrative statements such as provenance etc., are stored separately.

(4)   Plugin-based extensions: Ontowiki offers a framework for developing plugin extensions.

The choice of Ontowiki directly affected the selection of the underlying Triplestore since Ontowiki provides a pre-configured connector to the Openlink Virtuoso data management system, which members of the ENVRI-KMS team already had experience with from previous projects. The open-source edition of Openlink Virtuoso ^31^ (Version 7.2.5.1) was therefore deployed for that purpose and configured for Ontowiki (and vice-versa).

### 4.2 Tools for ingesting knowledge

The population of knowledge bases can take different routes. On the one hand, existing collections of information can sometimes be transformed so that they can be "bulk" imported into the ENVRI-KMS, which includes rearrangements and mappings of existing collections of structured information but potentially also the extraction of structured content from unstructured sources such as free text, which is by no means an easy task considering the complexity in the natural language processing/understanding. On the other hand, it is usually possible to manually add ENVRI-KMS’s contents, "fact by fact". However, manual input can be slow, tedious, and error-prone if not supported by dedicated tools. In the context of the ENVRI-KMS, it should be possible to provide content in both ways.

As far as manual data entry is concerned, the system supports the creation of valid RDF data via custom HTML Web forms. They are dynamically created using the RDForms ^32^ Javascript library based on formal JSON descriptions of the underlying data model. This also includes the specification of constrained SPARQL queries for the dynamic retrieval of menu options to maintain consistent RDF relationships between the described entity and related terminology and other entities already stored in the ENVRI-KMS.

### 4.3 FAIRness status sharing and gap analysis

To improve the findability, accessibility, interoperability, and reusability of digital research objects for both researchers and machines, the ENVRI-KMS offers a FAIR assessment dashboard ^8^. It supports RIs by discovering gaps in FAIR principle implementation at the granularity of their repositories and the discovery of possible technology solutions to address such gaps. For instance, the FAIRness assessment of a particular RI can be modified to indicate whether the repository contains machine-readable provenance information. By selecting an RI, the user interface gives an overview regarding its FAIRness status and gap analysis.

### 4.4 Ontowiki as a knowledge management platform

OntoWiki is a free and open-source semantic wiki web application that serves as an ontology editor and a knowledge acquisition system. Additionally, Ontowiki is a suitable RDF data management platform. A test instance is configured ^33^ and slightly customized to use the ENVRI logo and display the ENVRI RSS news feed on the front page. It currently serves as a data gateway for the facts added via forms based on the FAIR assessment dashboard. Ontowiki was found to perform well as RDF "middleware" used to ingest data from the RDF forms.

### 4.5 Search Engine

**Figure 5.**
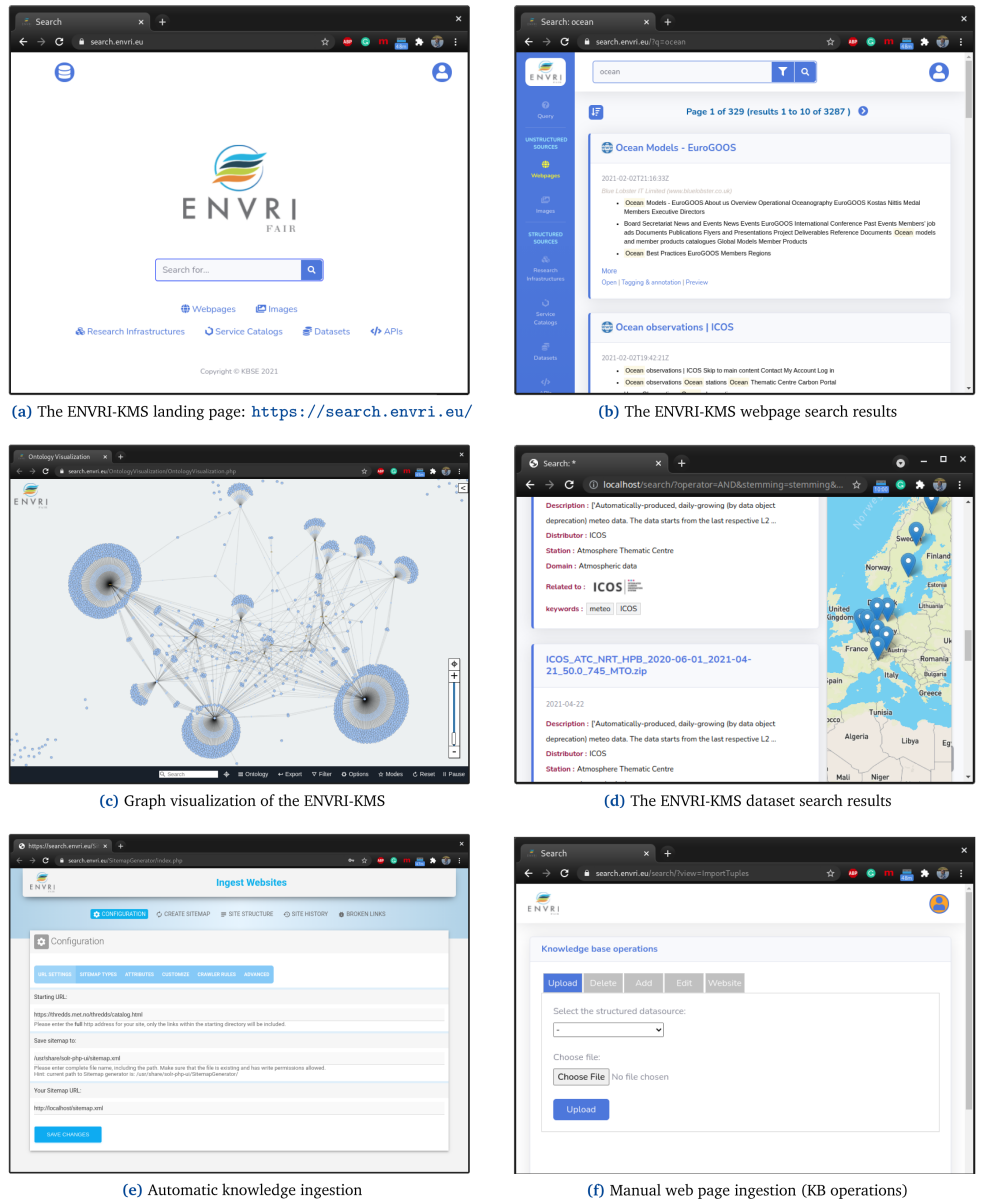
shows a set of functionality of the ENVRI-KMS.

In this section, we present a running example of the ENVRI-KMS Search Engine. To facilitate the general users to explore the ENVRI-KMS easily, we build the ENVRI-KMS Search Engine based on the Open Semantic Search’s fundamental concepts and components ^34^. Figure 5 illustrates the search interface ^9^.

A searcher can go to the landing page of the ENVRI-KMS (See Figure 5 (a)) directly and enter her search query in the search box and see the results immediately (See Figure 5 (b)). The results and their relevance to RIs and be visualized based on the graph visualization of the ENVRI-KMS (See 5 (c)). Note, the searcher can limit the search space of the ENVRI-KMS to a particular category, such as Webpages and RIs, as well. For instance, Figure 5 (d) shows the dataset search of the ENVRI-KMS.

The ENVRI-KMS can automatically capture, extract, and index knowledge regarding research assets based on the URL of the RIs (See Figure 5 (e)). Additionally, knowledge curators can ingest research assets manually to the knowledge base of the ENVRI-KMS (See Figure 5 (f)). Note, the ENVRI-KMS checks the indexed documents periodically to keep its knowledge base always up-to-date.

### 4.6 Operational workflow

**Figure 6.**
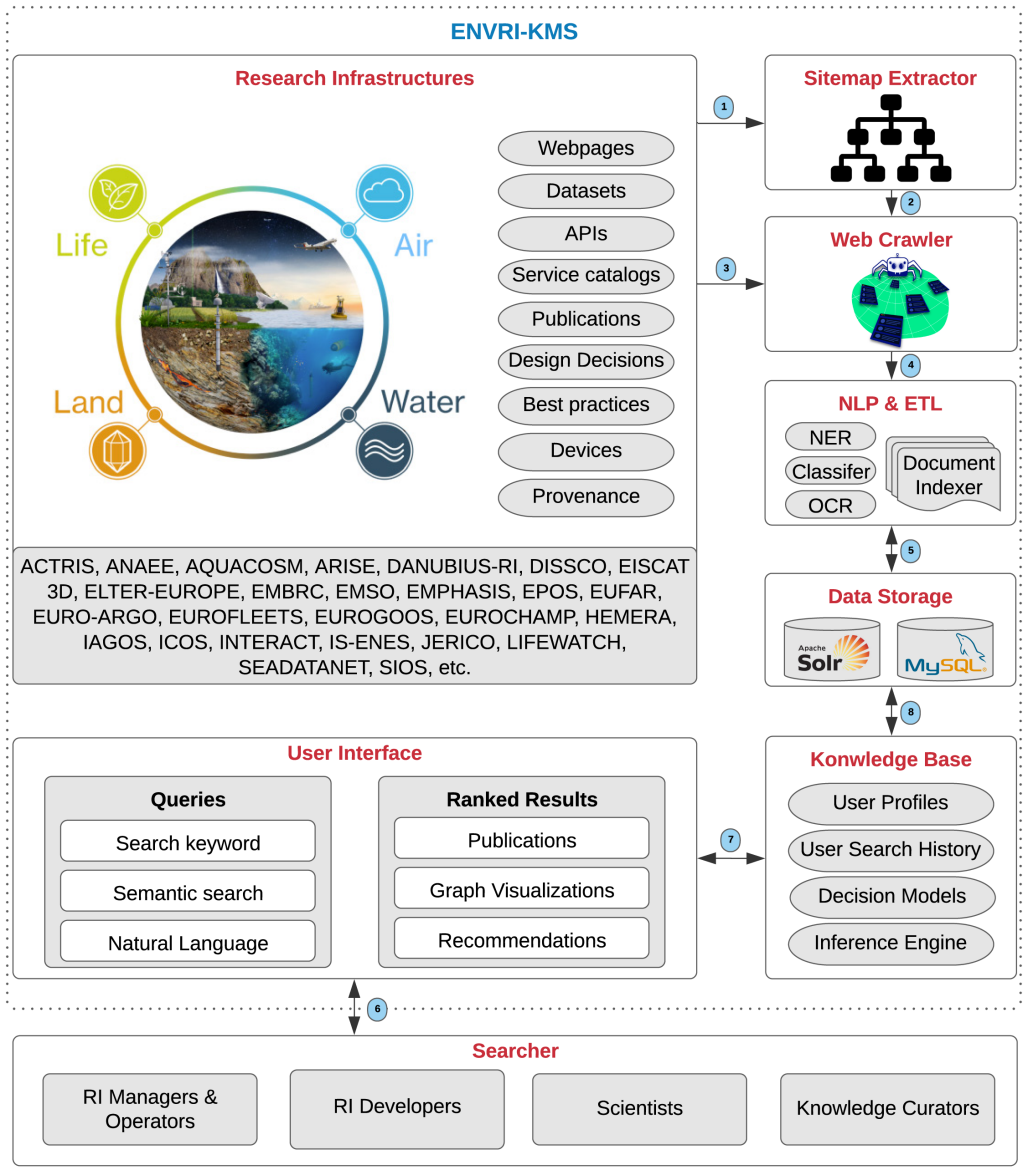
Represents the operational workflow of the ENVRI-KMS.

This section elaborates on the operational workflow of the ENVRI-KMS ^10^ and presents its constituent components (See Figure 6). **Research Infrastructures**, such as ACTRIS and ANAEE, are the primary sources of knowledge that contain knowledge assets, including webpages, datasets, APIs, service catalogs, publications, design decisions, best practices, devices, and data provenance. The **Sitemap Extractor** explores and extracts the site structure (the list of URLs) of the RIs. Then, the **Web Crawler** browses the extracted URLs and by employing **NLP & ETL** (Natural Language Processing and Extract/Transform/Load) techniques, such as Named-Entity Recognition (NER) and Relation Extraction (RE), tries to index documents and classify the extracted knowledge. Typically, a web crawler is a bot or software agent. It starts with a list of URLs to visit, called the seeds. As the crawler visits such URLs, it identifies all the hyperlinks on webpages by the aim of a sitemap extractor, and adds them to the list of its URLs to visit, called the crawl frontier. For instance, in the knowledge extraction process, the NER and RE approaches identify the entities represented in documents and their relations as fundamental knowledge extraction processes. The extracted knowledge is used to build the knowledge graph in the knowledge base of the ENVRI-KMS. **Data Storage** technologies, including Apache Solr and MySQL, are used to store the acquired knowledge systematically. The **Knowledge Base** of the ENVRI-KMS integrates user profiles, user search histories, decision models (e.g., meta-models), and infers solutions (results) based on searchers’ queries. the **User Interface** receives user queries, such as keywords and user stories, and demonstrated the results (e.g., publications, graph visualizations, and recommendations) to the **Searchers the process of extracting useful and hidden information 11 ** (See section 3.2).

## 5 Analysis

In this subsection, we reflect on each of the proposed design research questions based on our observations during the development process, the online survey, and documentation analysis.

### 5.1 Design decisions

We revisit the requirements and analyze the gap for the tools or platforms we investigated in terms of the requirements identified in Section 3.1.

**Compatible with Semantic Web technologies.** The two storage solutions (Apache Jena and Virtuoso) are triplestores dedicated to storing RDF data, thus fully meeting semantic web technology compatibility requirements. Regarding the knowledge management solutions, as the comparison in 30 indicates, both Semantic Mediawiki and Ontowiki are RDF compatible.

**Semantic search and query functionality.** Though the several Knowledge management systems investigated (like Ontowiki, Semantic Mediawiki) allow users to explore, search and edit the ENVRI-KMS’s content via GUI tools, they still lack easy user experience in terms of the technology required. The original purpose of both Semantic Mediawiki and Ontowiki is a semantic annotation of wiki pages and as a knowledge base editor, respectively.

**Open and flexible knowledge ingestion.** As shown in 30, knowledge management systems, such as Semantic Mediawiki and Ontowiki, support RDF import, facilitating the ingestion of knowledge. However, to prepare RDF triples or transform the information needed into knowledge, some customized tools needed to be designed and implemented considering the diversity of our project’s information sources.

**Provenance and version control of the knowledge.** As far as the considered knowledge management platforms are concerned, Ontowiki meets the requirements by providing detailed user management and statement-level provenance for RDF data, allowing tracking and potentially editing individual user contributions to the ENVRI-KMS.

**User-friendly and customizable user interface.** As already analyzed, although the Knowledge management systems provide a GUI for search and query, their targeted users are knowledge base administrators considering the technology barriers. For general users without much technical knowledge of the SPARQL or triplestores, a straightforward user interface for searching and exploration is expected to increase the user experience.

**Scaling and increasing performance.** Apache Jena Fuseki does not currently support horizontal scale-up, but there are workaround solutions like coordinating the updates from a staging server and publishing (read-only) to external clients. Based on the comparison, it is clear that no one single solution satisfies all the requirements. The optimal solution should be combining existing options, and other software such as Blazegraph could be a candidate.

### 5.2 Design research questions

To answer the first two design research questions ( *RQ*1 and *RQ*2), we have conducted an extensive literature review besides a set of expert interviews with domain experts at the RIs to build the search space (including webpages, datasets, etc.) of the ENVRI-KMS and capture knowledge systematically. The current search space ^11^ of the ENVRI-KMS includes all research infrastructures which are mentioned on the ENVRI community knowledge base ^12^. It is essential to highlight that the search space is not limited to the initial sets and grows automatically. Accordingly, the third design research question ( *RQ*3) can be addressed based on the natural language processing approach and Open Semantic Search that we have employed in the implementation of the ENVRI-KMS ^30^. To answer the fourth design research question ( *RQ*4), we have evaluated a set of technologies that can be employed to store and retrieve data. The last design research question ( *RQ*5) is one of the key challenges in this research. We plan to build a community around the ENVRI-KMS and ask the stakeholders, including domain experts, practitioners, and researchers, actively assess the search results and recommendations.

The FAIRness of the ENVRI-KMS should be elaborated in order to answer the study ’s main design research question. As a result, research assets become **Findable** when adequate metadata characterizes them and a searchable resource efficiently indexes them, allowing them to become recognized and available to potential users. A unique and persistent identifier should also be established so that the data may be referred and mentioned in research communications without ambiguity. The identifier facilitates data discovery and reuse by allowing persistent mapping between data, metadata, and other associated resources. The code or models required to utilise the data, research literature that provides additional insights into the data’s development and interpretation, and other related information are examples of related resources. The ENVRI-KMS indexes research assets and assigns them a unique identifier, allowing them to be shared among RIs.

**Accessibility** means that a human or a machine is given the exact conditions under which research assets can be accessed via metadata. Researchers in research communities can use the ENVRI-KMS to access research assets in accordance with RI policies and regulations.

The ENVRI-KMS search entities are characterized using normative and community-accepted specifications, vocabularies, and standards that define the precise meaning of concepts and qualities represented by the data. **Interoperability** is a crucial aspect of research assets’ value and usefulness. It is not only semantic interoperability that is important, but also technological and legal interoperability. Technical interoperability refers to the research assets being encoded using a standard that can be read by all systems involved.

The FAIR principles highlight the necessity for extensive metadata and documentation that match relevant community standards and give information about provenance in order for research materials to be reusable. The ability of humans and machines to evaluate and select research assets based on provenance information criteria is critical to their reuse. **Reusability** also necessitates the publication of research assets with a "clear and accessible usage license," which means that the terms under which the assets can be utilized should be transparent to both humans and machines.

Table 2 represents the mapping among the extracted requirements (R01 to R25) based on the responses of the participants to the survey questions (Q1 to Q5) and the design research question ( *RQ*1 to *RQ*5). Additionally, the table shows that more than half of the identified requirements (62% ) are at least partially addressed so that the main components of the ENVRI-KMS are functional.

**Table 2.** The mapping among the extracted requirements (R01 to R25) based on the responses of the participants to the survey questions (Q1 to Q5) and the design research question (RQ _1_ to RQ _5_). Additionally, the last column shows how far we have addressed the requirements up to now.

Requirements		Survey Questions					Research Questions					Addressed?
		*Q1*	*Q2*	*Q3*	*Q4*	*Q5*	*RQ1*	*RQ2*	*RQ3*	*RQ4*	*RQ5*	
*R01*	**Completeness of the ENVRI-KMS search space**	X				X	X					Partially
*R02*	**List of the contact persons**	X		X			X					Not yet
*R03*	**FAIRness criteria**	X	X		X						X	Yes
*R04*	**Entitiy types (private, open-source, etc.)**		X				X					Not yet
*R05*	**Recommendations**	X	X		X	X				X		Partially
*R06*	**Q&A forums for technical discussions**		X		X						X	Not yet
*R07*	**Ontologies and semantic search**		X			X				X		Partially
*R08*	**Multilingual queries**		X						X	X		Not yet
*R09*	**Source code and API search**		X							X		Not yet
*R10*	**Search RI website’s contents**		X	X						X		Yes
*R11*	**Standard user interface**			X		X				X		Yes
*R12*	**SPARQL queries**				X					X		Not yet
*R13*	**High performance and availability**				X					X		Partially
*R14*	**APIs to be connected to VREs**				X					X		Partially
*R15*	**Automatic knowledge ingestion**				X	X		X				Yes
*R16*	**Visualization**				X					X		Partially
*R17*	**Image search**				X	X				X		Partially
*R18*	**Assessment tools**		X		X	X					X	Not yet
*R19*	**Manual knowledge ingestion**				X			X				Yes
*R20*	**Updatable knowledge base**					X			X			Yes
*R21*	**Dataset geolocations**		X			X	X					Yes
*R22*	**Metadata of the search space entities**			X		X	X					Not yet
*R23*	**Continuous integration and continuous delivery**					X			X			Yes
*R24*	**Different user categories**					X					X	Partially
*R25*	**Categories & classifications**			X	X		X					Yes

## 6 Discussion

This section summarizes our observations and highlights several lessons learned during the development process of the ENVRI-KMS.

Software engineers have a broad knowledge of software development technologies, and they apply software engineering principles to develop software products. By employing such engineering principles in the software development lifecycle, from requirement analysis to software implementation and then deployment, they can build customized software products for individual stakeholders. The demand for highly skilled and qualified software engineers seems to have no end. This demand is growing in a changing economic landscape and fueled by the necessity of software development technologies. On the one hand, billions of dollars are spent annually on software products ^36^ that are produced and maintained by software engineers. On the other hand, business processes are introduced and managed by stakeholders and top-level managers who principally understand businesses ^37^.

Software architecture deals with the base structure, subsystems, and interactions among these subsystems, so it is critical to the success or failure of any software system ^38^. Software architecting can be thought of as a decision-making process in which software architects consider a collection of possible solutions for solving a system design problem and choose the one that is evaluated as the optimal ^39^. Software architecture decisions are design decisions that meet both functional and quality requirements in a system. Design decisions are concerned with the system’s application domain, architectural patterns employed in the system, Commercial off-the-shelf components, other infrastructure selections, and other aspects needed to satisfy all requirements ^40^. According to Avgeriou *et al.* ^41^, failing to make architectural design decisions during software development has well-known implications, such as costly system evolution, weak stakeholder communication, limited reusability of architectural assets, and poor traceability between specifications and implementation.

In order to make the design decisions to design the architecture of the ENVRI-KMS, we analyzed several alternative tools that could be used to build the fundamental components of the knowledge base. Selecting the right database system(s) (DBMS) was one of those design decisions. The DBMS selection problem is a subclass of the Commercial off-the-shelf (COTS) selection problem, and both problems are a subclass of Multi-Criteria Decision-Making (MCDM) problems ^42^. Accordingly, we used a decision support system that has been introduced by Farshidi *et al.* ^43^ to evaluate potential alternative solutions that we can employ to store and retrieve data. After performing an extensive evaluation, we decided to use *Apache Solr* to indexing the search entities and *MySQL* to manage user profiles and user search histories.

Judging the suitability of a set of technologies, such as programming languages, for developing a knowledge base system is a non-trivial task. For instance, a purely functional language like Haskell is the best fit for writing parallel programs that can, in principle, efficiently exploit huge parallel machines working on large data sets ^44^. However, while developing a dynamic website, a software engineer might consider ASP.net as the best alternative, and others might prefer using PHP or a similar scripting language. It is interesting to highlight that successful projects have been built with both: StackOverflow is built in ASP.net, whereas Wikipedia is built in PHP. Furthermore, a software engineer might prefer particular criteria, such as scalability in enterprise applications, whereas other criteria, such as technology maturity level, might have lower priorities.

We realized that we needed to select the right programming language ecosystems for developing the ENVRI-KMS. We used the decision model in the knowledge base of the decision support system ^45^ to evaluate potential programming languages that we can use to develop the ENVRI-KMS. Note, as mentioned earlier, we use an open-source tool, called open semantic search, in which its backend was implemented in *PHP* and *Python*, as the initial phase of the development process of the ENVRI-KMS. So that the first two solutions for us were these programming languages. However, the decision support system suggested *C#* , *Java*, and *Ruby* as three more alternative solutions. Finally, we decided to continue using Python, as we had more experience with it and found so many open-source projects on Github, which were implemented in Python, that could boost the development process.

Some issues were discovered regarding the cross-referencing of statements between knowledge bases (named graphs). A workaround published in a newsgroup provided a potential fix for static data but would have to be extended for a continuously growing data collection. A possible solution would be to store information that is expected to change/grow, e.g., the entity descriptions and the user terminology collected from the RDF forms, in a typical named graph and to configure Ontowiki filters for its efficient navigation while storing more static content, such as external ontologies, in separate graphs. While Ontowiki supports flexible navigation and data editing at the RDF statement level, the interface is arguably not appropriate for the vast majority of RI managers or developers. We conducted some experiments with the atmospheric domain, but RIs did not engage with the user interface. This is to be expected since Ontowiki relies on a good understanding of the RDF data model. Moreover, presenting information at the RDF statement’s granularity is typically inadequate for high-level information needs, e.g., discovering FAIR gaps in the data centers of an RI. We thus suggest that Ontowiki can act as an RDF-based middleware that powers high-level user applications and services. A critical aspect of using Ontowiki to manage the generated RDF data will be the question of versioning. While built-in features such as the statement-level provenance in principle allow detailed tracking of changes/revisions of the provided data, a backup strategy using external means should be considered as well. One straightforward step would be to export complete RDF dumps of the provided content in regular intervals and to track their versions in source code repositories such as Github.

## 7 Related work

In this research, Snowballing was the primary method to investigate the existing literature regarding tools and techniques that address the knowledge management challenges. A subset of selected studies is presented in Table 3.

**Table 3.** The results of the systematic literature review based on Snowballing (citation tracking) are presented here. The table shows the comparison of the selected studies and this study against a set of key factors, including research methods, publication types, research types, emphasized lifecycle phases, and contexts.

Study	Year	Research Method	Research Type	Lifecycle Phase	Context
This study	2021	Literature Study	Research Paper	Planning	Knowledge Engineering
		Document Analysis		Requirement Analysis	Knowledge Management
		Survey		Architecture Design	Knowledge Discovery
		Design Science		Implementation	Knowledge Acquisition
					Knowledge Representation
32	1992	Literature Study	Research Paper	Architecture Design	Knowledge Engineering Knowledge Acquisition
33	2001	Literature Study	Research Paper	Architecture Design	Decision-Making Process
34	2019	Literature Study	Research Paper	Planning	Knowledge Management
35	2018	Survey	Research Paper	Planning Requirement Analysis	Knowledge Management
36	2002	Literature Study	Research Paper	Planning	Knowledge Management
37	2005	Literature Study	Research Paper	Maintenance	Knowledge Management
38	2020	Literature Study Experiment	Research Paper	Architecture Design Implementation	Knowledge Discovery Knowledge Representation
39	2017	Literature Study	Research Paper	Planning	Knowledge Management
40	2019	Case Study	Research Paper	Planning	Knowledge Management
41	2019	Case Study	Research Paper	Planning	Knowledge Engineering Knowledge Management Knowledge Discovery
42	2018	Literature Study	Research Paper	Planning	Knowledge Management Decision-Making Process
43	2019	N/A	Tool Paper	Implementation	Knowledge Discovery
44	2017	Literature Study	Tool Paper	Architecture Design Implementation	Knowledge Discovery Knowledge Acquisition
13	2020	Literature Study Document Analysis Design Science	Tool Paper	Architecture Design Implementation	Decision-Making Process Knowledge Management
45	2018	Literature Study	Tool Paper	Architecture Design Implementation	Knowledge Discovery Knowledge Acquisition
46	2002	N/A	Tool Paper	Architecture Design Implementation	Knowledge Management
47	2006	Literature Study	Tool Paper	Architecture Design Implementation	Knowledge Management

Since 1990, business publications have started to publish an extensive list of research articles on knowledge management and decision support systems ^46^. Wielinga *et al.* ^47^ explained knowledge-based systems’ development as a modeling activity. Sapuan ^48^ reported a set of knowledge management systems’ architectures, concepts, and development processes. Additionally, the author highlighted the importance of knowledge-based systems in the context of concurrent engineering. Lee and Hong ^49^ define knowledge management concepts and distinguish them from business process reengineering and learning organization in terms of information technology application. Chau and Chuntian ^50^ proposed a knowledge management system on flow and water quality to simulate human expertise and heuristics in problem-solving and decision-making in the coastal hydraulic and transport processes. Akhavan *et al.* ^51^ explained and analyzed the main failure factors of implementing a knowledge management system in a pharmacist company. Park and Kim ^52^ proposed a framework for designing and implementing a knowledge management system for the fourth generation of Research and Development (R&D). Wachsmuth *et al.* ^53^ introduced a search engine framework for acquiring, mining, assessing, indexing, querying, retrieving, ranking, and presenting arguments while relying on standard infrastructure and interfaces. GIGGLE ^54^ is a genomics search engine that identifies and ranks the significance of genomic loci shared between query features and thousands of genome interval files. Santoro *et al.* ^55^ investigated the relationship among knowledge management systems, open innovation, knowledge management capacity, and innovation capacity. Farshidi *et al.* introduced a framework and knowledge management system to build decision models for database management systems ^56^, cloud service providers ^57^, software architecture patterns ^17, 58^, model-driven development platforms ^59^, programming languages ^45^ and blockchain platforms ^60^. Based on the literature study phase of the research, we realized that publications could be categorized into *knowledge management approaches* and *knowledge management systems*. There is a vast amount of literature about knowledge management approaches, such as ^47– 49, 51, 55^, 61– 66, that are research papers that mainly introduce methodologies that can be employed to discover, gather, manage, and apply a particular type of tacit or implicit knowledge. Alternatively, a significant number of tool papers can be found in literature ^17, 50, 52, 53, 57, 68^, that mainly present knowledge management systems that can be employed in a particular domain, such as software engineering or environmental science.

Additionally, we identified the phases of the software development life cycle (such as Planning, Requirement Analysis, and Architecture Design, Implementation) ^69, 70^ that selected studies have reported. During the literature study, we observed that the selected publications mainly researched within the following contexts: (1) **Knowledge Engineering** which emphasizes how to represent human knowledge (tacit knowledge) in a system and to extract interpretable information that can be turned into knowledge (explicit knowledge). (2) **Knowledge Management** that explains the process of creating, sharing, using, and managing the knowledge and information of an organization. (3) **Knowledge Discovery** that refers to the process of finding explicit knowledge in data and emphasizes the "high-level" application of particular data mining methods. The main goal is to extract such knowledge from data in the context of large databases. (4) **Knowledge Acquisition** which is the process used to define the rules and ontologies required for a knowledge-based system and is the process of extracting, structuring, and organizing knowledge from one source, usually human experts. (5) **Knowledge Representation** that translates information from the real world into a machine-understandable form and then utilizes acquired knowledge to solve complex decision-making problems. (6) **Decision-Making Process** which is a reasoning process based on assumptions of values, preferences, and beliefs of decision-makers. It leads to suggesting a set of solutions among several possible alternative options.

Table 3 shows the key factors of the selected studies and compares them against our study. The table shows that employed a combined research method that is based on literature study, document analysis, survey, and design science. Moreover, we reported the planning, requirement analysis, architecture design, and implementation phases of the ENVRI-KMS. The paper’s primary contexts are Knowledge Engineering, Knowledge Management, Knowledge Discovery, Knowledge Acquisition, and Knowledge Representation.

## 8 Conclusion and future Work

The development and operation of the ENVRI-KMS will be continuous. It will grow during the project while the development results and knowledge accumulate. The ENVRI-KMS development and operation depend on the development effort from the ENVRI subdomains and research infrastructures. The ENVRI-KMS should play a role in supporting developers from RIs to share best practices and find existing solutions, but the ENVRI community provides valuable input to the ENVRI-KMS and keeps it alive.

Currently, the ENVRI-KMS team closely interacts with the other subdomain developers (via workshops, meetings, and workgroups organized by subdomains). Through members, there is valuable input of the catalog of services, authentication and authorization, persistent identifier, triple store, license and usage tracking, and ENVRI-HUB.

The ENVRI-KMS team also closely interacts with semantic search workgroups in subdomains, e.g., a semantic search use case in ACTRIS ^13^ reported in the Semantic Search Working Group Final Report []FinalReport.

The ENVRI-KMS will continue in the rest of the ENVRI-FAIR project. In the next phase, the development effort will mainly focus on the following aspects: (1) Continuous content ingestion and curation. The ENVRI-KMS team will improve the knowledge ingestion tool and continuously ingest the description (metadata) of high-quality results from the ENVRI community (e.g., sub-domain or RI developers), including development results (e.g., best practices, software technologies, recommendations, updated FAIRness assessment possibly generated by new tools) in the ENVRI-KMS, and make those descriptions FAIR for the community.

(2)   Continuous improvement of the ENVRI-KMS based on the feedback is received from the community. Extra features, e.g., for ENVRI-KMS discovery and recommendation, will be further explored.

(3)   The development and operation of the ENVRI-KMS will also follow the software engineering DevOps practices. The continuous testing, integration, and deployment pipeline will be established.

(4)   We will also extend the content maintenance to community specialists. In this way, we hope the community will play a key role in the ENVRI-KMS.

## Data availability

### Underlying data

Mendeley Data: ENVRI-KMS. https://doi.org/10.17632/ntxypfsvds.1 ^21^

This project contains the following underlying data:

- Knowledgebase-discussion.xlsx (Raw survey outcome data)

### Extended data

- Analysis of the Survey: https://doi.org/10.17632/ntxypfsvds.1 ^21^
- Technology review, system design, documentation of the implementation, a demo of the ENVRI-KMS: https://doi.org/10.17632/n3khm4pnsd.1 ^30^

This project contains the following extended data:

- ENVRI-KMS (1).pdf (Summary of data analysis)
- Knowledgebase-discussion.pdf (Visualized survey outcomes)

Data are available under the terms of the Creative Commons Attribution 4.0 International license (CC-BY 4.0).

## Software availability

Software available from: SciCrunch: ENVRI-KMS,RRID:SCR_021235

Source code available from: https://github.com/SiamakFarshidi/solr-php-ui.git

Archived source code at time of publication: http://doi.org/10.5281/zenodo.4882766 ^19^

License: https://opensource.org/licenses/Apache-2.0

## Notes

1 Integrated Carbon Observation System (ICOS) ^7^

2 Creative Commons Attribution 4.0 international license (CC4BY)

3 In-service Aircraft for Global Observing System (IAGOS) ^8^

4 Aerosol, Clouds, and Trace Gases Research Infrastructure System (ACTRIS) ^9^

5 FAIR data are data that meet principles of findability, accessibility, interoperability, and reusability.

6 ENVRI-HUB is a one-stop-shop for access to environmental data and services provided by the contributing research infrastructures.

7 We have published the responses of the domain experts who participated in the survey besides the data analysis phases on Mendeley Data ^21^.

8 The FAIR dashboard can be accessed through the following link: https://envri-fair.github.io/knowledge-base-ui/

9 The ENVRI-KMS Search Engine is available through the following link: https://search.envri.eu

10 In order to implement the ENVRI-KMS, we have employed the proposed design decisions and solutions that Open Semantic Search ^34^ and WebVOWL ^35^ as two open-source projects have been offered.

11 https://search.envri.eu/

12 https://envri.eu/research-infrastructures/

13 https://github.com/xiaofengleo/actris
