# Supplementary material for: Knowledge sharing and discovery across heterogeneous research infrastructures
Source: Open Res Eur. 2023 Jun 6;1:68. Originally published 2021 Jun 14. [Version 3] doi: 10.12688/openreseurope.13677.3 (PMC10445897; doi:10.12688/openreseurope.13677.3)
Supplement: Peer review history

# Peer review: Reviewer response for version 3

**DOI:** 10.21956/openreseurope.17438.r32534

**Published:** 2023-06-12

**Authors:** Giacomo Marzi

**Affiliations:** 1 Department of Management, Mathematics and Statistics (DEAMS), University of Trieste, Trieste, Italy

Dear authors,

 Thank you for addressing all of my comments. From my perspective, the paper is now ready for indexing.

 I would also like to express my appreciation for your submission to Open Research Europe.

 Best wishes,

 GM

Is the rationale for developing the new software tool clearly explained?

Yes

Is the description of the software tool technically sound?

Yes

Are sufficient details of the code, methods and analysis (if applicable) provided to allow replication of the software development and its use by others?

Yes

Is sufficient information provided to allow interpretation of the expected output datasets and any results generated using the tool?

Partly

Reviewer Expertise:

innovation management, knowledge management

I confirm that I have read this submission and believe that I have an appropriate level of expertise to confirm that it is of an acceptable scientific standard.

# Peer review: Reviewer response for version 2

**DOI:** 10.21956/openreseurope.15455.r30062

**Published:** 2022-09-09

**Authors:** Giacomo Marzi

**Affiliations:** 1 Department of Management, Mathematics and Statistics (DEAMS), University of Trieste, Trieste, Italy

Dear authors,

 Thanks for your submission to Open Research Europe. Since I have stepped in during the second round of revision of the present paper, I will ground my comments even on the previous reviews and the previous reviewers’ comments.

 As a first impression, I have noticed a strong improvement on the paper, especially about setting the boundary of your study on ENVironmental Research Infrastructures (ENVRI), while I see additional room for improvement in some aspects of the paper.

 In particular, there are two points that require an additional revision, also considering the previous reviewers’ comments:

- It is still not clear the representativity of the sample and the details of the participants. You stated you conducted an online webinar with experts while not providing details about the questions, the topic of discussion, and the approach to the interviews.
  At the same time, you did not disclose any information about the participant in the subsequent online survey that is the core of your study.
  I would suggest a table (or some text) detailing the sample and how the sample has been selected.
- The second focal point to be addressed is about the practical implications. In Section 6, you aim to summarize the lesson learned during the ENVRI-KMS project. However, a practitioner could find it difficult to apply such findings or could be in difficult to find them inside the paper. As a result, I suggest adding a table summarizing in few bullet points the main implications of your study.

Thanks for your time and good luck with the review.

Is the rationale for developing the new software tool clearly explained?

Yes

Is the description of the software tool technically sound?

Yes

Are sufficient details of the code, methods and analysis (if applicable) provided to allow replication of the software development and its use by others?

Yes

Is sufficient information provided to allow interpretation of the expected output datasets and any results generated using the tool?

Partly

Reviewer Expertise:

innovation management, knowledge management

I confirm that I have read this submission and believe that I have an appropriate level of expertise to confirm that it is of an acceptable scientific standard, however I have significant reservations, as outlined above.

# Peer review: Reviewer response for version 2

**DOI:** 10.21956/openreseurope.15455.r28067

**Published:** 2021-12-06

**Authors:** Robert Huber

**Affiliations:** 1 Agricultural Economics and Policy AECP, Swiss Federal Institutes of Technology Zurich ETHZ, Zürich, Switzerland

The authors have significantly improved the manuscript by restructuring and condensing the previous draft.

Is the rationale for developing the new software tool clearly explained?

Partly

Is the description of the software tool technically sound?

Partly

Are sufficient details of the code, methods and analysis (if applicable) provided to allow replication of the software development and its use by others?

No

Is sufficient information provided to allow interpretation of the expected output datasets and any results generated using the tool?

Partly

Reviewer Expertise:

geosciences, environmental sciences, data management

I confirm that I have read this submission and believe that I have an appropriate level of expertise to confirm that it is of an acceptable scientific standard.

# Peer review: Reviewer response for version 1

**DOI:** 10.21956/openreseurope.14751.r27110

**Published:** 2021-07-12

**Authors:** Rebecca Koskela

**Affiliations:** 1 Ronin Institute, Montclair, NJ, USA; 2 University of New Mexico, Albuquerque, NM, USA

The paper summarizes the process for creating the Knowledge Management System for the Environmental Research Infrastructures (ENVRI) community.

**Introduction:**

- Assertions in this section require citations and they are missing. For example, "Due to population growth and economic development, human impacts on natural resources are continuing to grow." It also does not introduce the reader to ENVRI.

**Section 2:**

- This section is supposed to include the formulation of research questions. It doesn't accomplish this - the questions are generic and the connection to ENVRI is not obvious. Table 1 only adds confusion. Why would a KMS for mechanical engineering or product complaints be relevant? More discussion is needed for the choices in Table 1.

**Section 3:**

- Describes the survey that was used to gather requirements. It would be helpful to understand what breakout of the 35 participants, especially with respect to Figure 1. Was there sufficient participation by all the identified stakeholders? If there are 26 RIs, is 35 sufficient to discover the requirements?
- In addition, the acronym "KB" is used in Section 4 but not defined. It is most likely a typo. Another issue is the organization of the paper. The survey is discussed in Section 4 and then again in Section 6.

It would be interesting to read more about what is different about a knowledge management system for environmental science.

Is the rationale for developing the new software tool clearly explained?

Partly

Is the description of the software tool technically sound?

Partly

Are sufficient details of the code, methods and analysis (if applicable) provided to allow replication of the software development and its use by others?

No

Is sufficient information provided to allow interpretation of the expected output datasets and any results generated using the tool?

Partly

Reviewer Expertise:

High-performance computing; data management, bioinformatics

I confirm that I have read this submission and believe that I have an appropriate level of expertise to state that I do not consider it to be of an acceptable scientific standard, for reasons outlined above.

# Peer review: Reviewer response for version 1

**DOI:** 10.21956/openreseurope.14751.r27111

**Published:** 2021-06-24

**Authors:** Robert Huber

**Affiliations:** 1 Agricultural Economics and Policy AECP, Swiss Federal Institutes of Technology Zurich ETHZ, Zürich, Switzerland

The authors present a new knowledge management system to be used for environmental research infrastructures. Overall, this is certainly an innovative approach to make subject-specific knowledge and technical expertise easily findable and available also outside the own community. Unfortunately, the paper in its current form still has significant shortcomings, so that I would suggest indexing only after a fundamental revision and renewed review. 

 In detail, the paper leaves too much room for technology reviews and generalities. On the other hand, many questions remain open concerning the implementation and use of the tool. Many descriptions of the individual components are unfortunately still fragmentary and incoherent. In addition, a profound discussion is missing, which explains, for example, what distinguishes the proposed tool in comparison with commercial solutions and how the individual components and their interaction (given the identified user requirements and use cases) represent added value for the community here.

**Abstract**

- typo: ENVironmental

**Introduction**

 Para 1: 

- Would recommend not put population growth at the first place. Developped countries are main drivers.

Para 2: 

- IODE comment on data echange does not really fit here. (Instead a sentence on the necessity of observation systems would be required.

Para 5: 

- Avoid acronym CC4BY
- Expand IAGOS and ACTRIS acronyms
- Focus on atmosphere should be explained

Para 6:

- Please explain type of missing connects , assume you mean more fields than just data ?
- Please explain shortly what kind of information shall be captured? 

**Section 2:**

 Para 1: 

- Please differentiate between research environment and RI 
- First sentence seems to be broken 'such as ICOS are IAGOS'

Para 2:

-  These are not research questions but design questions,

Para 3:

- Para could imho be moved down, maybe also Para 1. Introduce the ENVRI-KMS then explain the methodology.

Para 5:

- I would say KMS is not covering every aspect of FAIR.
- Sure you mean development not operating and consuming communities in 'Knowledge-as-a-Service for the RI development communities.'
- Are these listed requirements a result of the methods described above? Maybe you should differentiate between community specific and generic requirements

**Section 3**

- Table 1:  explain terms used in the table to classify
- Section lacks an overview on existing commercial knowledge management systems.

**Section 3.1 and 3.2**

- Should be condensed and shortened and maybe moved to Introduction.
- The purpose of this sections is unclear to me. You describe literature covering 3 decades of research. Maybe you should extract a short, historically organised overview on past achievements instead of list every paper individually (which is already done in the Table).
- What are the consequences, conclusions of the lists described?

**Section 4.1**

- Please condense and shorten this section.

Para 1: 

- Online event?
- Here you mention 26 RI but you only introduced three above.
- Could you really select experts out of a larger pool?
- 'Firstly, we introduced the potential functionality' -> How did you get there? Is this the result of the literature analysis?

Para 2:

- At least the first few requirements sound as if they have been translated into some jargon. Are these the original reqs defined by RI experts?
- A bullet point list would be easier to read the whole section probably could be replaced by a table or appendix.
- Sentence broken: '(R01) have all potential RIs,'
- Define 'search space entity.'
- R03: please rewrite.

Para 3:

- So all asked RI experts were from the data domain? Please explain the background of experts above.
- User stories are not explained at all above this is however of key importance to understand the scope of the paper.

**Section 4.2**

 Move section up and explain use cases in more detail e.g. explain the focus on data (see comment above)

 (2) 

- Hard to understand, please rewrite.
- Update the state of their RI? Rather : get updated on ...
- Assessment wizard tool is not explained.
- Explain acronym KB.

(3) 

- Check which technologies? Implemented in RI or existing outside?
- Please rewrite this section what e.g. means 'prepared for FAIRness gaps'?

Figure 2: figure caption missing,  

**Section 4.4**

- Please strongly shorten and focus on the technologies chosen. You need not justify this by such a detailed technology overview.
- Consider to skip this section, the selected technologies are mentioned below anyway.

**Section 5**

 Instead of 'prioritized user stories' you should mention the use case you considered (data management etc.)

**Section 5.1**

 'It was suggested to consider OntoWiki' -> Did you choose this tool or another one?

**Section 5.3**

- This section is hard to understand and needs to be rewritten. It is not clear if this functionality is already in place or still planned.
- Links given as footnotes lead to binder linked notebooks which are not very useful to demonstrate how FAIR assessments are performed.

**Section 5.4**

- 'via forms based on the FAIRness analysis' -> This is not explained before.
- 'Some issues were discovered' which ones, is this important to describe the tool in this paper? Sections dealing with bug fixing are not necessary.
- Would suggest to move the section dealing with user engagement to the discussion.

**Section 5.5**

- This very short section suddenly jumps into document management issues.
- Please explain a bit how this was done, I assume here Elasticsearch was used?
- It is hard to understand the connection with Ontowiki is there any?

**Section 5.6**

- Web Crawler and Sitemap extractor are mentioned but not explained. In comparison to the lengthy description of OntoWiki this is not sufficient.
- Also sections on NLP etc. this would be more interesting in comparison to the tool selection parts above.
- Please expand and explain how this relates to Ontowiki and Search engine.

**Section 6.1**

 Many of the arguments listed here are already mentioned in the previous sections. Please merge and shorten (a lot) and ideally move this above/prior the technology selection section.

**Section 6.2**

- Literature: is this what has been selected to feed the system? Content is not very clear to me.
- Overall this section is much too long and a bit hard to follow, maybe this can be merged with some other section. The FAIR principles are not helpful in this context to describe how research questions have been addressed.

**Section 7**

 Please do not discuss 'Software architecture' and Implementation in general, instead discuss the architecture and implementation of the ENVRI-KMS. The whole section needs to be rewritten in way that it focuses on the ENVRI KMS.

**General comment**

 Usually KM systems also care a lot about 'tacit knowledge' but I assume you rather deal with documents and data?

Is the rationale for developing the new software tool clearly explained?

Partly

Is the description of the software tool technically sound?

Partly

Are sufficient details of the code, methods and analysis (if applicable) provided to allow replication of the software development and its use by others?

No

Is sufficient information provided to allow interpretation of the expected output datasets and any results generated using the tool?

Partly

Reviewer Expertise:

geosciences, environmental sciences, data management

I confirm that I have read this submission and believe that I have an appropriate level of expertise to state that I do not consider it to be of an acceptable scientific standard, for reasons outlined above.
